# Statistical optimization of chitosan-based synthesis strategies to generate albumin nanoparticles

**DOI:** 10.1007/s13346-026-02046-4

**Published:** 2026-02-16

**Authors:** Aitana Martín-Escaño, Claudia Barbuzano, Juan Manuel Rodríguez-Díaz, Edgar Pérez-Herrero

**Affiliations:** 1 Grupo de Investigación de Encapsulación y Evaluación Biológica Avanzada (ENCAPBIO-ULL Research Group), Departamento de Ingeniería Química y Tecnología Farmacéutica, Universidad de Laguna. Avda. Astrofisico Francisco Sánchez, S/N, 38206 San Cristóbal de La Laguna (Santa Cruz de Tenerife), Spain; 2 Escuela de Doctorado y Estudios de Posgrado, Universidad de La Laguna. Avda. Astrofísico Francisco Sánchez, 38206 La Laguna (Santa Cruz de Tenerife), Spain; 3 Departamento de Estadística, Instituto Universitario de Física Fundamental y Matemáticas, Universidad de Salamanca, 37008 Salamanca, Spain

**Keywords:** Nanoparticles, Albumin, Chitosan, Design of experiments (DoE), Optimization

## Abstract

Albumin-based nanoparticles (NPs) are typically synthesized by harsh conditions-based methods that limit their application in clinics and can seriously damage the entrapped drug and even their base material. Despite the potential of the use of chitosan (CS) as stabilizing agent by adapting the ionic gelation method or by adding CS as a coating to albumin NPs generated by desolvation, the influential factors of these methods have not yet been studied. In this article, these synthesis approaches have been optimized by a 2-step DoE-based methodology (a screening process with fractional designs plus a response surface methodology using central composite designs). The application of the ion gelation method to produce albumin-based NPs generates sizes from 66 to 1017 nm, PDI (polydispersity index) values of 0.3–0.6 and surface charges (ZP) from neutral to positive (> 20 mV). The fitted models of the responses depend on four factors (albumin and CS concentration, CS pH and CS:albumin mass ratio). On the other hand, the modification of the desolvation method using CS as a stabilizing coating generates 37–1305 nm NPs, with PDI between 0.4 and 0.7 and highly positive ZP (20–40 mV). In this case, the approximate models for the responses depend on four main effects (albumin and CS concentration, pH of CS and albumin:EtOH volume ratio). Furthermore, in this work the best combinations of factors and levels that allow minimizing PDI and obtaining the minimum and maximum expected values for mean size and ZP of NPs were determined for both synthesis methods. Focusing on the minimum possible PDI, the predicted values for the ion gelation- and desolvation-based methods are 0.363 and 0.341, respectively, which are achieved with values of [BSA] (mg/ml), [CS] (mg/ml), CS pH and CS:BSA or BSA:EtOH ratios (mL:mL) of {2.3,1.4,2.2,1:7.3} and {10,0.5,1.8,1:1}, respectively. These optimized conditions yield acceptable size and ZP values for the ion gelation-based (27.7 nm; 16.4 mV) and optimal values for the desolvation-based (146.2 nm; 29.5 mV).

**Graphical abstract:**

**Figure:**
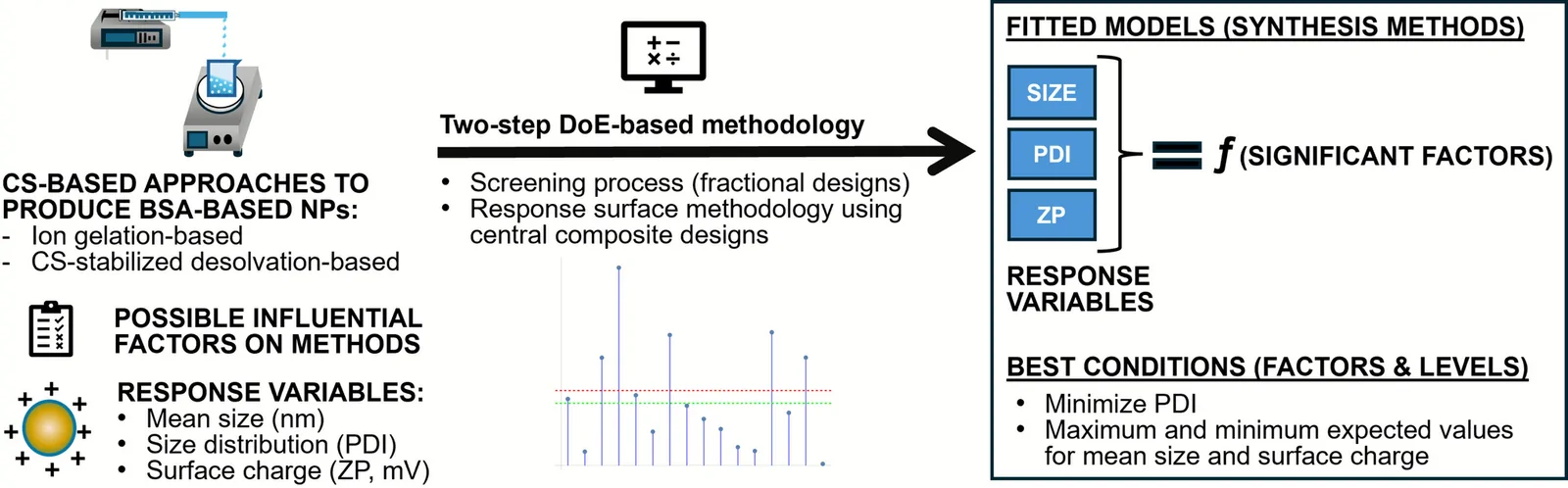

**Supplementary information:**

The online version contains supplementary material available at https://doi.org/10.1007/s13346-026-02046-4.

## Introduction

Protein-based nanoparticles (NPs), particularly those implying serum albumin, have been widely used as carriers of different therapeutic molecules and drugs for the treatment of multiple diseases as they present an extraordinary biocompatibility with a complete absence of immunogenicity and toxicity, a high stability in vitro and in vivo and a preeminent biodegradability with harmless degradation products when metabolized in vivo [1–3]. Albumin-based NPs can passively target cell surface receptors, such as the glycoprotein 60 (GP60 or albondin) that is expressed on vascular endothelial cells or the secreted protein acidic and rich in cysteine (SPARC or osteonectin) that is overexpressed in tumor cells or intestinal inflammation [4, 5]. Moreover, the binding ability of albumin to SPARC can be used to cross the blood brain barrier to treat neurodegenerative diseases [6, 7] and brain tumors [8]. Apart from GP60 and SPARC, there are other promising albumin binding proteins [9], such as the neonatal Fc receptor (FCRN) in cancer [10] or Megalin and Cubilin that expand the therapeutic application of the albumin-based NPs to kidney-related diseases [11, 12]. Additionally, these nanocarriers possess multiple binding sites that facilitate the incorporation of multiple drug types in their matrix, adding the possibility of their inclusion by electrostatic interactions due to the albumin primary structure [1]. Thus, there are already two albumin-based NPs on the market, Abraxane® (albumin-bound paclitaxel NPs or nab™ paclitaxel), approved by FDA in 2005 for metastatic breast cancer and in 2012–2013 for non-small cell lung cancer and metastatic pancreatic cancer [13] and Fyarro® (nab™ sirolimus) approved by FDA (2021) for metastatic malignant perivascular epithelial cell tumors (PEComa) [14, 15].

Albumin-based NPs can be prepared by different methods, mainly desolvation [16–20], emulsification-based [21–25], thermal gelation [26–29] or self-assembly [30–33]. Although desolvation method is the most used due to its simplicity and reproducibility, the resulting NPs need to be stabilized using crosslinking agents, mainly glutaraldehyde, of known toxicity [34–36]. The emulsification-based techniques use organic solvents and high-shear homogenization, while the self-assembly procedures use reducing or denaturant agents with potential toxicity and heating treatments. Similarly, thermal gelation techniques employ temperatures that are not appropriate for heat-sensitive molecules [37, 38]. Clearly, these harsh conditions can limit the in vivo application of albumin-based NPs, compromise the integrity of their cargo and cause the albumin to lose or reduce its benefits, which is leading to the search for new synthetic strategies. In this regard, the group of Zhao first reported the use of the ion gelation method to synthesize albumin-based NPs by reacting the positively charged chitosan in acidic pH with the negatively charged albumin in basic pH [39, 40]. Moreover, several authors, based on the work of the group of Uludag [41, 42], have reported the use of chitosan as an alternative to glutaraldehyde to stabilize the albumin NPs generated by the desolvation method, avoiding the toxicity of the possible residual content of cross-linking agents [43–48]. However, despite the considerable interest of these methods, since they only utilize albumin and chitosan, a polysaccharide with bio/muco-adhesive properties that allow interactions with the mucosal barriers, and do not require the use of any severe synthetic condition, the effects of the variables of these synthesis processes on the physicochemical properties of NPs have not yet been studied. Therefore, in this work, a two-step design of experiments (DoE) methodology has been conducted to optimize these processes of synthesis of albumin-based NPs, where chitosan is an essential component of the formulation. The factors that affect the process were detected and their effects were determined, obtaining correlations between factors and responses and the optimal conditions of synthesis. All the information obtained from this article will be fundamental for the clinical development of these nanosystems to facilitate the passive delivery of bioactive molecules to tumor tissues and inflamed areas (e.g. inflammatory bowel disease) and improve their accumulation, and enable them to cross the blood–brain barrier to achieve effective treatments against neurodegenerative diseases, which are currently based solely on symptomatic approaches.

## Materials and methods

### Materials

Bovine serum albumin (BSA) and low molecular weight chitosan (CS) with a deacetylation degree greater than 75% were provided by Sigma-Aldrich (Germany). Dulbecco’s Phosphate Buffered Saline (DPBS), acetic acid (glacial) and ethanol absolute were also obtained from Sigma-Aldrich (Germany).

### Synthesis of albumin-based nanoparticles by the ion gelation method

**Fig. 1:**
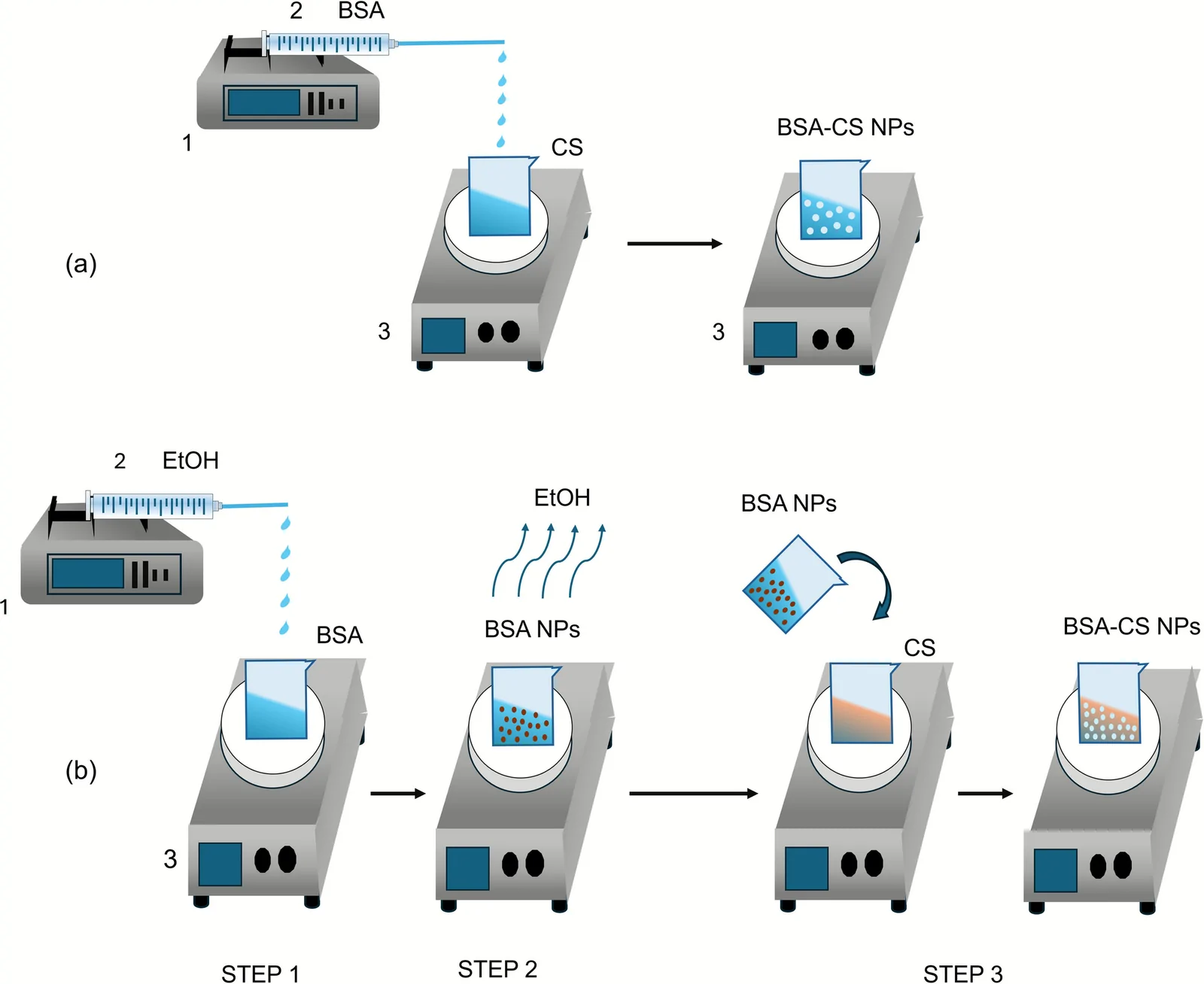
Schemes of the synthetic procedures to generate BSA-based nanoparticles. (**a**) Adaptation of the ion gelation method first described by Hou et al. [39] to generate the BSA-CS_ie NPs. (**b**) Adaptation of the modified desolvation method first reported by Han et al. [43]. Step 1: Desolvation process to generate BSA NPs; Step 2: Evaporation of ethanol; Step 3: Stabilization of the BSA NPs by a CS coating to generate the BSA-CS_dsolv NPs. (1) Syringe pump; (2) Syringe with BSA solution or EtOH; (3) Magnetic stirrer

Albumin NPs were prepared by the adaptation of the ion gelation method, first described by Hou et al. [39] to obtain albumin-based NPs and which is based on the electrostatic interaction of the positively charged CS with the negatively charged albumin at basic pH. Briefly, an aqueous solution of BSA in DPBS (1 or 10 mg/mL) at an adjusted basic pH (7.3 or 9.3) was added under magnetic agitation at room temperature with a syringe pump (Legato 110, Kd Scientific, USA) at a flow rate of 0.5 or 2.0 mL/min into a 0.5 or 3 mg/mL aqueous CS solution in acetic acid with acidic pH (1.7 or 3). The respective volumes of both solutions were adjusted to reach mass ratios CS:BSA of 1:4 or 1:8. Once solutions were mixed, magnetic stirring was maintained for 10 min, obtaining BSA-CS NPs (BSA-CS_ie NPs). A scheme of the procedure is shown in Fig. 1a.

### Synthesis of chitosan-stabilized albumin-based nanoparticles

Albumin NPs were prepared by an adaptation of the modified desolvation method that was first reported by Han et al. [43] and uses chitosan as an alternative to glutaraldehyde. Briefly, first, different volumes of ethanol (EtOH) were added over an aqueous solution of BSA (10 or 80 mg/mL) with an adjusted pH (5.1 or 9.1) until 1:1 or 1:3 EtOH:BSA volume ratios were achieved. This first step was done under agitation at room temperature with a syringe pump (Legato 110, Kd Scientific, USA) at a flow rate of 0.5 or 2.0 mL/min to cause BSA to phase separate and aggregate into nanoparticles (BSA NPs) that need further stabilization. Magnetic stirring was maintained until total evaporation of EtOH and then BSA NPs suspension was added under agitation into an aqueous CS solution (0.5 or 3 mg/mL) in acetic acid with acidic pH (1.7 or 2.5) to stabilize them with a CS coating, formed by electrostatic interaction around the negatively charged surface of the previously formed BSA NPs. After that, stirring was maintained for another 20 min to complete the coating process, obtaining chitosan-stabilized BSA NPs (BSA-CS_dsolv NPs). A scheme of the procedure is shown in Fig. 1b.

### Characterization of the albumin-based nanoparticles

Preparations were characterized in terms of size and polydispersity by dynamic light scattering (DLS) and surface charge by electrophoretic light scattering (ELS) (Zetasizer Nano ZS, Malvern Panalytical, England) in the SEGAI (Servicio General de Apoyo a la Investigación) of the Universidad de La Laguna.

### Experimental doe design and statistical analysis

Both synthesis processes were statistically optimized by a two-step DoE procedure. A multiresponse model was considered with size, polydispersity index (PDI) and surface charge (Z Potential, ZP) as the response variables.

Initially, the factors with possible influence in these synthetic processes were selected by an extensive literature review and multiple preliminary experiments were performed to fix the levels of the factors, using particle formation as the general criteria. Then, in order to identify the factors with more influence in the considered response variables, a screening procedure was performed using a main-effect model, including just the intercept and the first order factors in the model, without interaction terms, dealing with few parameters and, thus, needing less observations. For this, 2_IV_^8−4^ and 2_IV_^7−3^ fractional designs, with maximum resolution and minimum aberration, were used for the ion gelation-based method and the desolvation-based method with CS stabilization, respectively. All the factors were considered to have just two levels, −1 (low) and + 1 (high), to reduce the number of experiments. These setups were chosen over the Plackett–Burman designs following the specialized literature [49] that warns about the confusion between main effects and two-factor interactions when using the Plackett–Burman designs, which may produce problems when deciding about the significant effects.

After the screening process, the remaining factors and their powers and interactions were analyzed using Response Surface Methodology (RSM) techniques to obtain approximate second-order models, relating the factors with the response variables (size, PDI and ZP). A third level (central or ‘0’ in coded variable), which is the mean of the levels of each factor, was considered for all factors to take central observations, which are later used to check model curvature. To apply the Steepest Ascent Method, a first-order polynomial model was proposed initially, and later, for the location of the stationary points, a more complex model, including interaction and curvature terms, was proposed to fit approximately the response variables. Central Composite Designs (CCD), that is, designs with factorial, central and axial points, were employed, taking observations for several combinations of the factor levels.

Analysis of Variance (ANOVA) methodology, which focuses on decomposing the total data variability in different parts and identifying each one with a specific source, has been employed to detect factors influence in the models considered in each phase. The parameters of the final models were estimated employing the least squares method, performing t-test on them to determine the significant ones and decide whether they should be included in the model or not.

## Results and discussion

### Synthetic approaches to generate albumin-based nanoparticles by using CS

The main aim of this work is to optimize two alternative synthetic procedures to obtain BSA-based NPs without using harsh conditions, in such a way that the cargo and the albumin itself are not damaged and lose their bioactive properties. Thus, two previously reported methods that use CS as the only complementary material to BSA have been adapted, being, therefore, the concentration and pH of BSA and CS factors with a possible effect on the physicochemical properties of the NPs. Note that since BSA and Human Serum Albumin (HSA) share about 80% of sequence and structure homology and similar biological functions (e.g. maintenance of osmotic pressure or transport of small molecules) [50], BSA was used in this work, considering its lower cost, abundance and safety, which supports its routine use for standard non-clinical research [51]. In fact, BSA-based NPs are a reasonable model for initial screening. However, the use of HSA will be mandatory for the next phases of development of these NPs due to the absence of immunogenicity of HSA [2, 50, 52], something essential for the clinical translation of this drug delivery nanosystem. In addition, as size, polydispersity and drug encapsulation and release of HSA-based NPs may differ from that of BSA-based NPs [53], in vivo behavior should be validated with HSA-based NPs.

In the particular case of the ion gelation-based method, the procedure requires only the electrostatic interaction between CS and BSA through the addition of the protein to the polymer under agitation, so the flow rate of BSA and the stirring conditions, as well as the CS:BSA mass ratio, seem to be factors with possible influence in the process to be considered. Then, in this method of synthesis, eight factors with possible influence on the process can be identified. Table 1 shows these factors and their levels. Regarding the desolvation method with CS stabilization, since the only modification with respect to the conventional one is the substitution of glutaraldehyde by CS as the stabilizer, the addition flow rate of EtOH over the BSA solution, the BSA:EtOH volume ratio and the stirring rate of the CS solution during the addition of the BSA NPs suspension to it, can be considered as additional factors that influence the process. Thus, in this method, seven factors have a possible influence on the generation of the NPs, which are included in Table 2, along with their levels. Note that before stabilizing the BSA NPs with CS, EtOH is removed by evaporation, a process that was monitored by controlling initial and final volumes in the samples. This is the standard procedure found in literature [54, 55] since EtOH is a Class 3 solvent with low toxicity and residual quantities of EtOH are accepted without justification in nanomedicines [56]. Solvent removal would be a critical issue if solvents involved in the synthesis are Class 2, in which case their removal must be assessed, typically, by gas chromatography-based techniques [56–59].

**Table 1:** Experimental factors and their levels considered for the ion gelation-based method (BSA-CS_ie NPs)

Levels	[BSA] (mg/mL)	pH BSA	[CS] (mg/mL)	pH CS	CS:BSA (mg:mg)	BSA FR (mL/min)	SR (during addition, rpm)	SR (post addition, rpm)
*−1*	1.0	7.3	0.5	1.7	1:4	0.5	300	300
+ *1*	10.0	9.3	3.0	3.0	1:8	2.0	600	600

*BSA*: Bovine Serum Albumin; *CS*: chitosan; *FR*: flow rate; *SR*: stirring rate; *(−1)*: low level; *(+ 1)*: high level

**Table 2:** Experimental factors and their levels of the desolvation-based technique (BSA-CS_dsolv NPs)

Levels	[BSA] (mg/mL)	pH BSA	[CS] (mg/mL)	pH CS	BSA:EtOH (mL:mL)	EtOH FR (mL/min)	SR (rpm)
*−1*	10.0	5.1	0.5	1.7	1:1	0.5	300
+ *1*	80.0	9.1	3.0	2.5	1:3	2.0	600

*BSA*: Bovine Serum Albumin; *CS*: chitosan; *FR*: flow rate; *SR*: stirring rate; *(−1)*: low level; *(+ 1)*: high level

For both synthesis methods, the levels of the factors were set as distant as possible (Tables 1 and 2), but allowing for the correct formation of the NPs, based on preliminary experiments and a literature review. Note that the ranges for the levels were initially established based on an extensive literature review and subsequently fixed through experimental studies, considering all factors and employing particle formation as the principal criterion. Outside the ranges presented in Table 1, the occurrence of visible precipitates and/or micron-scale aggregates were observed.

Although both synthesis methods allowed obtaining particles of very different sizes by varying the levels of the factors considered, a PDI value below 0.4 was not achieved in most cases, this being a response to be optimized. As can be seen in Table 3, the size obtained with the ion gelation-based method varied significantly from 66 to 1017 nm, when using the levels of the factors of Table 1, with PDI values ranging from 0.3 to 0.6 in most cases. Regarding the particles obtained with the desolvation method with CS stabilization, the range of sizes that can be achieved, with the values of factors indicated in Table 2, is wider (37—1305 nm) and with a shift in the mean PDI towards values between 0.4 to 0.7 (Table 4). The surface charge (ZP) of the NPs is positive regardless of the method used, with significantly higher values being obtained with the desolvation-based method (Tables 3 & 4). Note that although many preliminary experiments were done to define the actual levels for the factors (assuring the correct generation of the ALG-CS NPs), in Tables 3 and 4 were included only those experiments at the final levels of the factors.

**Table 3:** Responses (Size, PDI & ZP) as function of factors for the ion gelation-based method (BSA-CS_ie NPs)

	Factors and their values								Responses		
	*[BSA] (mg/mL)*	*pH BSA*	*[CS] (mg/mL)*	*pH CS*	*CS:BSA (mg:mg)*	*BSA FR (mL/min)*	*SR (rpm)*		Size(nm)	PDI	ZP(mV)
							^^	**			
***1***	1.0	7.3	0.5	1.7	1:4	2.0	600	600	260.8	0.355	14.2
***2***	1.0	7.3	0.5	1.7	1:8	2.0	600	600	148.4	0.442	12.3
***3***	1.0	7.3	0.5	3.0	1:4	2.0	600	600	168.7	0.702	19.1
***4***	1.0	7.3	0.5	3.0	1:8	2.0	600	600	292.8	0.821	9.3
***5***	1.0	7.3	3.0	1.7	1:4	2.0	600	600	571.3	0.572	18.9
***6***	1.0	7.3	3.0	1.7	1:8	2.0	600	600	86.3	0.379	12.9
***7***	1.0	7.3	3.0	3.0	1:4	2.0	600	600	175.8	0.324	5.1
***8***	1.0	7.3	3.0	3.0	1:8	2.0	600	600	167.0	0.399	1.1
***9***	1.0	9.3	0.5	1.7	1:4	2.0	600	600	452.1	0.562	16.7
***10***	1.0	9.3	0.5	1.7	1:8	2.0	600	600	359.7	0.443	7.6
***11***	1.0	9.3	0.5	3.0	1:4	2.0	600	600	356.2	0.464	17.2
***12***	1.0	9.3	0.5	3.0	1:8	2.0	600	600	341.7	0.489	11.3
***13***	1.0	9.3	3.0	1.7	1:4	2.0	600	600	189.4	0.331	18.6
***14***	1.0	9.3	3.0	1.7	1:8	2.0	600	600	150.5	0.373	11.5
***15***	1.0	9.3	3.0	3.0	1:4	2.0	600	600	406.6	1.000	0.8
***16***	1.0	9.3	3.0	3.0	1:8	2.0	600	600	739.6	0.716	0.7
***17***	10.0	7.3	0.5	1.7	1:4	2.0	600	600	400.4	0.628	19.2
***18***	10.0	7.3	0.5	1.7	1:8	2.0	600	600	402.0	0.739	23.3
***19***	10.0	7.3	0.5	3.0	1:4	2.0	600	600	66.3	0.224	21.1
***20***	10.0	7.3	0.5	3.0	1:8	2.0	600	600	153.1	0.560	19.1
***21***	10.0	7.3	3.0	1.7	1:4	2.0	600	600	446.5	0.643	16.2
***22***	10.0	7.3	3.0	1.7	1:8	2.0	600	600	289.9	0.427	20.7
***23***	10.0	7.3	3.0	3.0	1:4	2.0	600	600	237.5	1.000	17.6
***24***	10.0	7.3	3.0	3.0	1:8	2.0	600	600	1017.0	0.914	4.9
***25***	10.0	9.3	0.5	1.7	1:4	2.0	600	600	265.5	0.514	17.7
***26***	10.0	9.3	0.5	1.7	1:8	2.0	600	600	659.5	0.678	18.3
***27***	10.0	9.3	0.5	3.0	1:4	2.0	600	600	69.3	0.492	26.2
***28***	10.0	9.3	0.5	3.0	1:8	2.0	600	600	376.0	1.000	13.8
***29***	10.0	9.3	3.0	1.7	1:4	2.0	600	600	427.6	0.668	9.2
***30***	10.0	9.3	3.0	1.7	1:8	2.0	600	600	237.2	0.490	18.3
***31***	10.0	9.3	3.0	3.0	1:4	2.0	600	600	347.9	0.774	12.0
***32***	10.0	9.3	3.0	3.0	1:8	2.0	600	600	789.5	0.412	5.7

*BSA* bovine serum albumin, *CS* chitosan, *FR* flow rate, *SR* stirring rate (^^: during addition of BSA over CS, **: after the addition process), *PDI* polydispersity index, *ZP* Z Potential

**Table 4:** Responses (Size, PDI & ZP) as function of factors for desolvation-based method (BSA-CS_dsolv NPs)

	Factors and their values							Responses		
	*[BSA] (mg/mL)*	*pH BSA*	*[CS] (mg/mL)*	*pH CS*	*BSA:EtOH (mL:mL)*	*EtOH FR (mL/min)*	*SR (rpm)*	Size (nm)	PDI	ZP (mV)
***1***	10.0	5.1	0.5	1.7	1:1	2.0	600	358.4	0.488	35.5
***2***	10.0	5.1	0.5	1.7	1:3	2.0	600	622.5	0.280	22.9
***3***	10.0	5.1	0.5	2.5	1:1	2.0	600	304.0	0.500	42.8
***4***	10.0	5.1	0.5	2.5	1:3	2.0	600	278.5	0.964	44.4
***5***	10.0	5.1	3.0	1.7	1:1	2.0	600	976.6	0.746	33.6
***6***	10.0	5.1	3.0	1.7	1:3	2.0	600	946.2	0.745	31.6
***7***	10.0	5.1	3.0	2.5	1:1	2.0	600	223.9	0.864	43.8
***8***	10.0	5.1	3.0	2.5	1:3	2.0	600	489.8	0.791	30.6
***9***	10.0	9.1	0.5	1.7	1:1	2.0	600	317.7	0.471	32.5
***10***	10.0	9.1	0.5	1.7	1:3	2.0	600	489.4	0.609	29.8
***11***	10.0	9.1	0.5	2.5	1:1	2.0	600	113.8	0.579	31.3
***12***	10.0	9.1	0.5	2.5	1:3	2.0	600	476.0	0.614	34.8
***13***	10.0	9.1	3.0	1.7	1:1	2.0	600	549.4	0.528	32.1
***14***	10.0	9.1	3.0	1.7	1:3	2.0	600	909.4	0.475	26.0
***15***	10.0	9.1	3.0	2.5	1:1	2.0	600	315.1	0.919	37.1
***16***	10.0	9.1	3.0	2.5	1:3	2.0	600	357.0	0.689	42.7
***17***	80.0	5.1	0.5	1.7	1:1	2.0	600	516.9	0.584	18.6
***18***	80.0	5.1	0.5	1.7	1:3	2.0	600	1305.0	0.668	20.3
***19***	80.0	5.1	0.5	2.5	1:1	2.0	600	148.5	0.620	19.5
***20***	80.0	5.1	0.5	2.5	1:3	2.0	600	256.1	0.583	25.7
***21***	80.0	5.1	3.0	1.7	1:1	2.0	600	784.1	0.867	22.9
***22***	80.0	5.1	3.0	1.7	1:3	2.0	600	1593.0	0.344	16.3
***23***	80.0	5.1	3.0	2.5	1:1	2.0	600	44.2	0.662	27.5
***24***	80.0	5.1	3.0	2.5	1:3	2.0	600	771.3	0.406	14.4
***25***	80.0	9.1	0.5	1.7	1:1	2.0	600	599.7	0.619	21.8
***26***	80.0	9.1	0.5	1.7	1:3	2.0	600	1179.0	0.575	17.8
***27***	80.0	9.1	0.5	2.5	1:1	2.0	600	61.7	0.796	21.5
***28***	80.0	9.1	0.5	2.5	1:3	2.0	600	316.8	0.600	20.6
***29***	80.0	9.1	3.0	1.7	1:1	2.0	600	702.3	0.775	21.3
***30***	80.0	9.1	3.0	1.7	1:3	2.0	600	838.1	0.645	17.1
***31***	80.0	9.1	3.0	2.5	1:1	2.0	600	37.8	0.700	24.0
***32***	80.0	9.1	3.0	2.5	1:3	2.0	600	514.9	0.587	26.4

*BSA* bovine serum albumin, *CS* chitosan, *EtOH* ethanol, *FR* flow rate, *SR* stirring rate, *PDI* polydispersity index, *ZP* z potential

### Two-step doe statistical analysis and optimization

#### Screening methodology

As stated above, a screening procedure was initially performed in order to identify the factors of both synthesis methods with more influence in the physicochemical properties of the BSA-CS NPs (that is, the response variables, size, PDI and ZP) without performing too many observations. Tables 5 and 6 include these fractional designs employed and the values of the responses for both synthesis methods, noting that the experiments were performed in a random order. To employ all the possible information, the experiments that were performed initially (Tables 3 & 4) were also used in this screening procedure.

**Table 5:** Design matrix for the 2_IV_^8−4^ fractional design used in the ion gelation-based method

	Factors								Responses (BSA-CS_ie NPs)		
	x[1]	x[2]	x[3]	x[4]	x[5]	x[6]	x[7]	x[8]	Size (nm)	PDI	ZP (mV)
**y**[1]	−1	−1	−1	−1	−1	−1	−1	−1	192.4	0.444	14.9
**y**[2]	+ 1	−1	−1	−1	−1	+ 1	+ 1	+ 1	634.8	0.728	23.0
**y**[3]	−1	+ 1	−1	−1	+ 1	−1	+ 1	+ 1	173.4	0.374	12.8
**y**[4]	+ 1	+ 1	−1	−1	+ 1	+ 1	–1	−1	640.9	0.686	23.5
**y**[5]	−1	−1	+ 1	−1	+ 1	+ 1	+ 1	−1	74.6	0.378	10.2
**y**[6]	+ 1	−1	+ 1	−1	+ 1	−1	−1	+ 1	231.0	0.485	16.9
**y**[7]	−1	+ 1	+ 1	−1	−1	+ 1	−1	+ 1	275.2	0.372	16.5
**y**[8]	+ 1	+ 1	+ 1	−1	−1	−1	+ 1	−1	482.9	0.701	16.3
**y**[9]	−1	−1	−1	+ 1	+ 1	+ 1	−1	+ 1	195.0	1.000	10.1
**y**[10]	+ 1	−1	−1	+ 1	+ 1	−1	+ 1	−1	88.0	0.294	18.2
**y**[11]	−1	+ 1	−1	+ 1	−1	+ 1	+ 1	−1	342.5	0.533	10.9
**y**[12]	+ 1	+ 1	−1	+ 1	−1	−1	−1	+ 1	175.9	0.251	27.7
**y**[13]	−1	−1	+ 1	+ 1	−1	−1	+ 1	+ 1	314.0	0.952	4.0
**y**[14]	+ 1	−1	+ 1	+ 1	−1	+ 1	−1	−1	248.6	1.000	26.1
**y**[15]	−1	+ 1	+ 1	+ 1	+ 1	−1	−1	−1	431.5	0.782	−0.4
**y**[16]	+ 1	+ 1	+ 1	+ 1	+ 1	+ 1	+ 1	+ 1	754.8	0.886	5.8

x[1]: BSA concentration (mg/mL); x[2]: BSA pH; x[3]: CS concentration (mg/mL); x[4]: CS pH; x[5]: CS:BSA mass ratio; x[6]: BSA flow rate (mL/min); x[7] stirring rate during addition of BSA over CS (rpm); x[8]: stirring rate post addition (rpm); PDI: polydispersity index; ZP: Z Potential (surface charge); y[1]-y[16]: number of runs

**Table 6:** Design matrix for the 2_IV_^7−3^ fractional design used in the desolvation method with CS stabilization

	Factors							Responses (BSA-CS_dsolv NPs)		
	x[1]	x[2]	x[3]	x[4]	x[5]	x[6]	x[7]	Size (nm)	PDI	ZP (mV)
**y**[1]	−1	−1	−1	−1	−1	−1	−1	366.6	0.285	26.9
**y**[2]	+ 1	−1	−1	−1	+ 1	−1	+ 1	1244.0	0.427	31.2
**y**[3]	−1	+ 1	−1	−1	+ 1	+ 1	−1	484.8	0.802	31.1
**y**[4]	+ 1	+ 1	−1	−1	−1	+ 1	+ 1	776.1	0.669	21.4
**y**[5]	−1	−1	+ 1	−1	+ 1	+ 1	+ 1	939.7	0.950	30.5
**y**[6]	+ 1	−1	+ 1	−1	−1	+ 1	−1	840.3	0.806	22.1
**y**[7]	−1	+ 1	+ 1	−1	−1	−1	+ 1	914.9	0.554	41.1
**y**[8]	+ 1	+ 1	+ 1	−1	+ 1	−1	−1	1104.0	0.636	19.1
**y**[9]	−1	−1	−1	+ 1	−1	+ 1	+ 1	604.8	0.584	33.2
**y**[10]	+ 1	−1	−1	+ 1	+ 1	+ 1	−1	354.3	0.543	22.3
**y**[11]	−1	+ 1	−1	+ 1	+ 1	−1	+ 1	700.7	0.484	40.1
**y**[12]	+ 1	+ 1	−1	+ 1	−1	−1	−1	214.8	0.673	24.0
**y**[13]	−1	−1	+ 1	+ 1	+ 1	−1	−1	564.7	0.918	25.9
**y**[14]	+ 1	−1	+ 1	+ 1	−1	−1	+ 1	139.0	0.874	32.6
**y**[15]	−1	+ 1	+ 1	+ 1	−1	+ 1	−1	672.5	0.900	41.0
**y**[16]	+ 1	+ 1	+ 1	+ 1	+ 1	+ 1	+ 1	614.2	0.526	34.7

x[1]: BSA concentration (mg/mL); x[2]: BSA pH; x[3]: CS concentration (mg/mL); x[4]: CS pH; x[5]: BSA:EtOH volume ratio (mL:mL); x[6]: EtOH flow rate (mL/min); x[7] stirring rate (rpm); PDI: polydispersity index; ZP: Z Potential (surface charge); y[1]-y[16]: number of runs

The screening phase indicated that in the ion gelation-based method the last three factors (BSA flow rate and the stirring rates during and after the addition of BSA over CS) could be removed since the responses (Table 5) did not show significant differences between the levels of the factors (See Supplementary Material. Appendix). However, it should be noted that preliminary experiments (Table 3) provided little information for these three factors since all the experiments were taken at fixed values of them. Although, to our knowledge, there is not much literature about this method [39, 40, 60, 61], the results obtained are as expected [60, 61]. Note that this method is essentially an electrostatic interaction, something very common in the generation of NPs by complexation between alginate and CS, of which there are many examples in the literature [62–66].

Regarding the desolvation-based method, the results obtained in the screening phase, using the data from the fractional design (Table 6) of this step and those obtained from the previous experiments done at the same levels of the factors (Table 4), indicated that factors 2 and 6 (BSA pH and EtOH flow rate) were not significant and could be removed from the model (See Supplementary Material. Appendix). Considering the lack of literature on the stabilization of the generated BSA NPs with CS, these results are consistent with the existing literature on the conventional desolvation method, except for the BSA pH that usually has a large effect on particle size [16, 67, 68]. The lack of significance of this factor in this modified method may be due to the electrostatic interaction of the BSA NPs with CS, when the latter is used as a stabilizer instead of glutaraldehyde.

#### Response surface methodology

After the screening procedure, five factors remain in the models of both synthesis processes. At this point, a Response Surface Methodology (RSM) was applied to these factors to fit the models that approximate the response variables. A third level (central or ‘0’ in coded variable) is considered for all factors in order to check curvature. Tables 7 and 8 included this new central level in the significant factors, taking into account that for the discarded factors all the subsequent experiments were always carried out at the respective center levels.

**Table 7:** Natural and coded factors in the RSM procedure for the ion gelation-based method (BSA-CS_ie NPs)

*Levels*	*[BSA] (mg/mL)*	*pH BSA*	*[CS] (mg/mL)*	*pH CS*	*CS:BSA (mg/mg)*	*BSA FR (mL/min)*	*SR (during addition, rpm)*	*SR (post addition, rpm)*
*−1*	1.0	7.3	0.5	1.7	1:4	1.25	450	450
*0*	5.5	8.3	1.75	2.4	1:6			
+ *1*	10.0	9.3	3.0	3.0	1:8			

*BSA*: Bovine Serum Albumin; *CS*: chitosan; *FR*: flow rate; *SR*: stirring rate; *(+ 1)*: high level; *(0)*: center level; *(−1)*: low level

**Table 8:** Natural and coded factors in the RSM procedure for the desolvation method (BSA-CS_dsolv NPs)

*Levels*	*[BSA] (mg/mL)*	*pH BSA*	*[CS] (mg/mL)*	*pH CS*	*BSA:EtOH (mL/mL)*	*EtOH FR (mL/min)*	*SR (rpm)*
*−1*	10.0	7.1	0.5	1.7	1:1	1.25	300
*0*	45.0		1.75	2.1	1:2		450
+ *1*	80.0		3.0	2.5	1:3		600

*BSA*: Bovine Serum Albumin; *CS*: chitosan; *EtOH*: ethanol; *FR*: flow rate; *SR*: stirring rate; *(+ 1)*: high level; *(0)*: center level; *(−1)*: low level

Since the levels of the factors were taken as distant as possible, it was not possible to extend the range of them out of these limits and thus a cubical design region was employed. When applying the Steepest Ascent Method the curvature test turned out to be significant and thus full quadratic models were considered without moving from the center of the experimental region, and Central Composite Designs (CCD) were used for fitting the response variables (size, PDI and ZP) for both synthesis methods, always fixing the removed factors at the respective central level (Tables 7 and 8). Axial observations were additionally taken, two at each factor axis, usually at a distance between 1 and the square root of the number of active factors involved (5 in this case). In this case, given the cubical experimental region, the distance of the axial points cannot be greater than 1, thus their coordinates need to be 1 or −1 and therefore the designs are non-rotatable. The specific designs that were employed for fitting the response variables when using the ion gelation- and desolvation-based methods are shown in Tables 9 and 10, respectively, as well as the values of the responses for the experiments. In both designs, the first 32 runs correspond to the 2^5^ factorial design, the following five are the central observations taken at the center level of every factor and the last ten are the axial points that due to the cubical design region were taken at the faces of the design hypercube. All the experiments were performed in a random order.

**Table 9:** Central Composite Design used in the final fitting of the response variables for the ion gelation-based method and values of the responses of the BSA-CS_ie NPs

	Factors					Responses		
	x[1]	x[2]	x[3]	x[4]	x[5]	Size (nm)	PDI	ZP (mV)
**y**[1]	−1	−1	−1	−1	−1	94.9	0.512	13.9
**y**[2]	+ 1	−1	−1	−1	−1	215.2	0.424	19.5
**y**[3]	**−1**	+ 1	−1	−1	−1	157.1	0.359	12.9
**y**[4]	+ 1	+ 1	−1	−1	−1	275.1	0.416	15.7
**y**[5]	−1	−1	+ 1	−1	−1	240.0	0.382	12.6
**y**[6]	+ 1	−1	+ 1	−1	−1	371.5	0.534	22.6
**y**[7]	−1	+ 1	+ 1	−1	−1	146.2	0.261	17.5
**y**[8]	+ 1	+ 1	+ 1	−1	−1	405.2	0.606	19.7
**y**[9]	−1	−1	−1	+ 1	−1	331.3	0.373	18.4
**y**[10]	+ 1	−1	−1	+ 1	−1	42.4	0.336	16.5
**y**[11]	−1	+ 1	−1	+ 1	−1	436.6	0.358	15.0
**y**[12]	+ 1	+ 1	−1	+ 1	−1	50.1	0.277	21.2
**y**[13]	−1	−1	+ 1	+ 1	−1	300.5	0.484	18.2
**y**[14]	+ 1	−1	+ 1	+ 1	−1	237.0	0.766	21.6
**y**[15]	−1	+ 1	+ 1	+ 1	−1	155.6	0.536	2.5
**y**[16]	+ 1	+ 1	+ 1	+ 1	−1	281.0	0.973	19.1
**y**[17]	−1	−1	−1	−1	+ 1	112.3	0.331	15.1
**y**[18]	+ 1	−1	−1	−1	+ 1	432.4	0.573	18.0
**y**[19]	−1	+ 1	−1	−1	+ 1	166.9	0.320	15.5
**y**[20]	+ 1	+ 1	−1	−1	+ 1	874.0	0.487	16.7
**y**[21]	−1	−1	+ 1	−1	+ 1	104.2	0.287	14.4
**y**[22]	+ 1	−1	+ 1	−1	+ 1	161.7	0.414	21.5
**y**[23]	−1	+ 1	+ 1	−1	+ 1	84.6	0.244	15.1
**y**[24]	+ 1	+ 1	+ 1	−1	+ 1	282.7	0.434	16.8
**y**[25]	−1	−1	−1	+ 1	+ 1	131.9	1.000	10.1
**y**[26]	+ 1	−1	−1	+ 1	+ 1	75.0	0.309	15.9
**y**[27]	−1	+ 1	−1	+ 1	+ 1	566.1	0.652	9.1
**y**[28]	+ 1	+ 1	−1	+ 1	+ 1	190.4	0.488	19.9
**y**[29]	−1	−1	+ 1	+ 1	+ 1	68.0	0.657	6.7
**y**[30]	+ 1	−1	+ 1	+ 1	+ 1	1057.0	0.985	9.9
**y**[31]	−1	+ 1	+ 1	+ 1	+ 1	417.3	0.820	1.1
**y**[32]	+ 1	+ 1	+ 1	+ 1	+ 1	850.8	0.817	7.4
**y**[33]	0	0	0	0	0	85.1	0.472	20.6
**y**[34]	0	0	0	0	0	46.3	0.414	17.8
**y**[35]	0	0	0	0	0	37.8	0.349	17.0
**y**[36]	0	0	0	0	0	52.9	0.316	22.4
**y**[37]	0	0	0	0	0	34.9	0.374	18.6
**y**[38]	−1	0	0	0	0	96.2	0.366	17.1
**y**[39]	+ 1	0	0	0	0	56.7	0.391	19.8
**y**[40]	0	−1	0	0	0	110.3	0.277	15.5
**y**[41]	0	+ 1	0	0	0	141.7	0.331	17.8
**y**[42]	0	0	−1	0	0	163.0	0.250	27.6
**y**[43]	0	0	+ 1	0	0	23.6	0.354	10.7
**y**[44]	0	0	0	−1	0	258.8	0.398	18.7
**y**[45]	0	0	0	+ 1	0	106.5	0.988	7.7
**y**[46]	0	0	0	0	−1	150.7	0.264	17.6
**y**[47]	0	0	0	0	+ 1	25.3	0.403	20.9

x[1]: BSA concentration (mg/mL); x[2]: BSA pH; x[3]: CS concentration (mg/mL); x[4]: CS pH; x[5]: CS:BSA mass ratio (mg:mg); y[1]-y[47]: number of runs; −1: low level; + 1: high level; 0: center level. Note that in this design, experiments were performed using the center level (0) for the factors x[6]: BSA flow rate (mL/min), x[7]: stirring rate during addition of BSA over CS (rpm) and x[8]: stirring rate post addition (rpm)

**Table 10:** Central Composite Design used in the final fitting of the response variables for the desolvation-based method and values of the responses of the BSA-CS_dsolv NPs

	Factors					Responses		
	x[1]	x[3]	x[4]	x[5]	x[7]	Size (nm)	PDI	ZP (mV)
**y**[1]	−1	−1	−1	−1	−1	298.6	0.482	22.8
**y**[2]	+ 1	−1	−1	−1	−1	850.4	0.612	16.3
**y**[3]	−1	+ 1	−1	−1	−1	797.4	0.437	30.6
**y**[4]	+ 1	+ 1	−1	−1	−1	947.0	0.673	18.1
**y**[5]	−1	−1	+ 1	−1	−1	256.6	0.476	46.1
**y**[6]	+ 1	−1	+ 1	−1	−1	68.7	0.625	18.8
**y**[7]	−1	+ 1	+ 1	−1	−1	202.5	0.844	36.6
**y**[8]	+ 1	+ 1	+ 1	−1	−1	103.4	0.893	12.9
**y**[9]	−1	−1	−1	+ 1	−1	650.0	0.565	28.6
**y**[10]	+ 1	−1	−1	+ 1	−1	1046.0	0.973	21.8
**y**[11]	−1	+ 1	−1	+ 1	−1	1684.0	0.828	23.5
**y**[12]	+ 1	+ 1	−1	+ 1	−1	741.0	0.861	21.0
**y**[13]	−1	−1	+ 1	+ 1	−1	270.4	0.852	39.7
**y**[14]	+ 1	−1	+ 1	+ 1	−1	218.2	0.569	24.8
**y**[15]	−1	+ 1	+ 1	+ 1	−1	534.7	0.706	38.3
**y**[16]	+ 1	+ 1	+ 1	+ 1	−1	301.1	0.546	16.1
**y**[17]	−1	−1	−1	−1	+ 1	207.7	0.566	11.6
**y**[18]	+ 1	−1	−1	−1	+ 1	869.5	0.624	9.4
**y**[19]	−1	+ 1	−1	−1	+ 1	543.1	0.471	31.0
**y**[20]	+ 1	+ 1	−1	−1	+ 1	723.6	0.766	16.9
**y**[21]	−1	−1	+ 1	−1	+ 1	189.3	0.604	46.6
**y**[22]	+ 1	−1	+ 1	−1	+ 1	35.1	0.802	18.0
**y**[23]	−1	+ 1	+ 1	−1	+ 1	536.5	0.800	33.6
**y**[24]	+ 1	+ 1	+ 1	−1	+ 1	99.2	0.992	28.4
**y**[25]	−1	−1	−1	+ 1	+ 1	416.4	0.776	27.5
**y**[26]	+ 1	−1	−1	+ 1	+ 1	1005.0	0.645	18.6
**y**[27]	−1	+ 1	−1	+ 1	+ 1	852.0	0.836	29.2
**y**[28]	+ 1	+ 1	−1	+ 1	+ 1	792.0	0.784	22.3
**y**[29]	−1	−1	+ 1	+ 1	+ 1	642.6	0.513	34.0
**y**[30]	+ 1	−1	+ 1	+ 1	+ 1	108.2	0.571	16.0
**y**[31]	−1	+ 1	+ 1	+ 1	+ 1	421.6	0.825	24.6
**y**[32]	+ 1	+ 1	+ 1	+ 1	+ 1	301.7	0.573	15.6
**y**[33]	0	0	0	0	0	258.0	0.884	24.5
**y**[34]	0	0	0	0	0	268.2	0.924	25.6
**y**[35]	0	0	0	0	0	341.6	0.702	23.9
**y**[36]	0	0	0	0	0	274.5	0.974	38.0
**y**[37]	0	0	0	0	0	339.9	0.713	28.0
**y**[38]	−1	0	0	0	0	789.0	0.707	48.0
**y**[39]	+ 1	0	0	0	0	223.7	0.915	27.4
**y**[40]	0	−1	0	0	0	241.6	0.585	23.7
**y**[41]	0	+ 1	0	0	0	288.4	0.905	18.3
**y**[42]	0	0	−1	0	0	723.2	0.851	24.9
**y**[43]	0	0	+ 1	0	0	122.6	0.992	19.2
**y**[44]	0	0	0	−1	0	299.3	0.684	20.2
**y**[45]	0	0	0	+ 1	0	361.8	0.920	30.8
**y**[46]	0	0	0	0	−1	601.7	0.933	30.6
**y**[47]	0	0	0	0	+ 1	238.4	0.818	41.2

*x[1]* BSA concentration (mg/mL), *x[3]* CS concentration (mg/mL), *x[4]* CS pH, *x[5]* BSA:EtOH volume ratio (mL:mL), *x[7]* stirring rate (rpm), *y[1]-y[47]* number of runs, *−1* low level, *+ 1* high level, *0* center level. Note that in this design, experiments were performed using the center level (0) for the factors x[2]: BSA pH and x[6]: EtOH flow rate (mL/min)

Second-order polynomials were fitted by the least squares method for both synthesis techniques. Below are the results of this process for both the ion gelation- and the desolvation-based synthesis methods, and for the three response variables (PDI, size and surface charge or ZP).

## Ion gelation-based synthesis method (BSA-CS_ie NPs)

### Model for the response variable “PDI”

Of the two synthesis procedures reported in this work, the one based on the ion gelation method is the one that allows obtaining lower values of PDI, although most combinations of factors yield values between 0.3 and 0.6, with those below 0.4 being acceptable for having monodisperse suspensions of nanoparticles, so the minimization of this response variable is of great interest. Note that obtaining a narrow size distribution is critical to obtain stable, reproducible and controllable carriers with predictable drug release profiles [69].

**Fig. 2:**
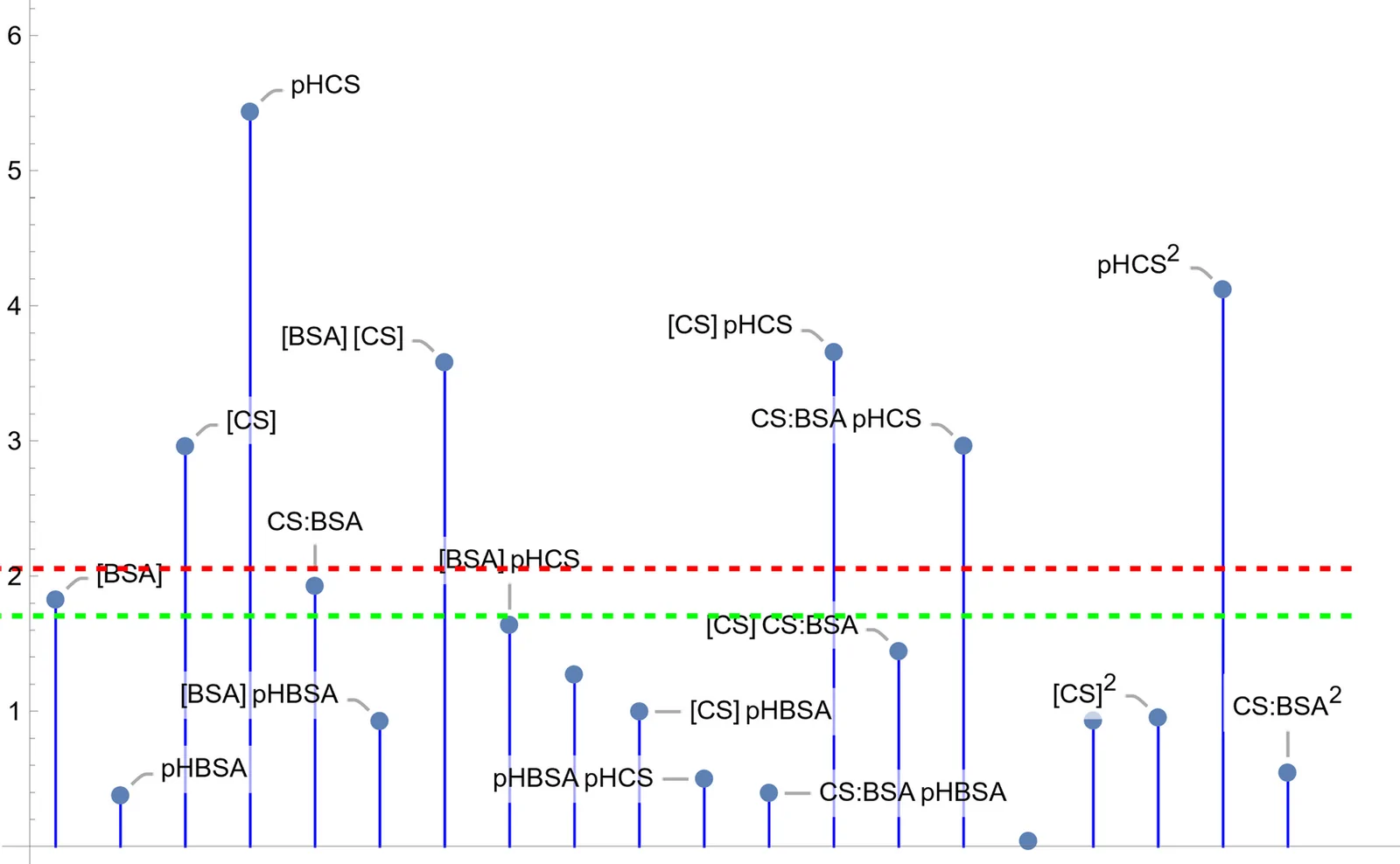
Parameter t-test absolute values for the PDI model (ion-gelation method). Values over the green line are significant at 10% significance level, those above the red line are significant for 5% level of significance

From the parameter tests of significance (Fig. 2), it is clear that CS has a significant effect on PDI, both its pH ‘pHCS’ and its concentration ‘[CS]’. Moreover, since the p-value for both [BSA] and CS:BSA mass ratio is between 5 and 10% and there are significant interactions involving them, i.e. ‘[BSA] [CS]’ and ‘CS:BSA pHCS’, it seems convenient to include them in the model. Unfortunately, these results cannot be confirmed with the limited existing literature on this method, except for the effect of CS:BSA [61], nor even using that corresponding to NPs generated by alginate-CS complexation. There are no other effects in a similar situation, so the final fitted model for PDI contains 4 main effects, 3 two-factor interactions and a pure quadratic term, as it is stated in Eq. (1), which explains the 71.3% of the data variability (R^2^ = 0.713, Adjusted R^2^ = 0.652).

1 $$PDI=5.309-0.0171\;\left[BSA\right]-0.0974\; CS:BSA-0.258\;\left[CS\right]-3.967 \;pHCS+0.0136\;\left[BSA\right]\;\left[CS\right]+0.0495 \;CS:BSA\; pHCS+0.0979\;\left[CS\right]pHCS+0.775\;{pHCS}^{2}$$

where ‘[BSA]’ refers to BSA concentration, ‘[CS]’ to CS concentration, ‘CS:BSA’ to CS:BSA mass ratio and ‘pHCS’ to CS pH.

When looking for factor combinations that minimize PDI, several options can be considered, noting that the design space has been chosen to be the cubical region [−1,1]x[−1,1]x[−1,1]x[−1,1]x[−1,1] since out of this region the conditions for the experiments are not guaranteed. Thus, there is an ‘internal’ solution for the five factors, which is a solution not in the borders (edges or corners), {−0.716, F2, −0.258,−0.246,0.643} in coded variables (where ‘F2’ in the second component of the vector means that the response value does not depend on the second factor and, thus, the most convenient value between −1 and 1 could be used for it), with an expected value of 0.363 for the response. Moreover, there are, as well, other options that allow to obtain lower expected PDI values (around 0.320), for instance {−1,1,1,−0.428,1} or {−1,1,−1,−0.0265,−1}, but in the borders of the design region, which could be non-convenient since an extreme value for any of the factors means that the point is just in the border of the ‘valid’ region where the conditions are more stressed. Thus, the best proposal seems to be the first one, which in natural variables can be expressed as {2.278, 8.310, 1.428, 2.213, 1:7.286}, taking the second factor (the non-influential) at the center value. That is: [BSA] = 2.28 mg/ml; [CS] = 1.43 mg/ml, pHCS = 2.2, CS:BSA = 1:7.29 (mg/mg), and the remaining factors are fixed at the central levels. As stated before, the expected PDI value for these conditions would be 0.363.

### Model for the response variable “size”

**Fig. 3:**
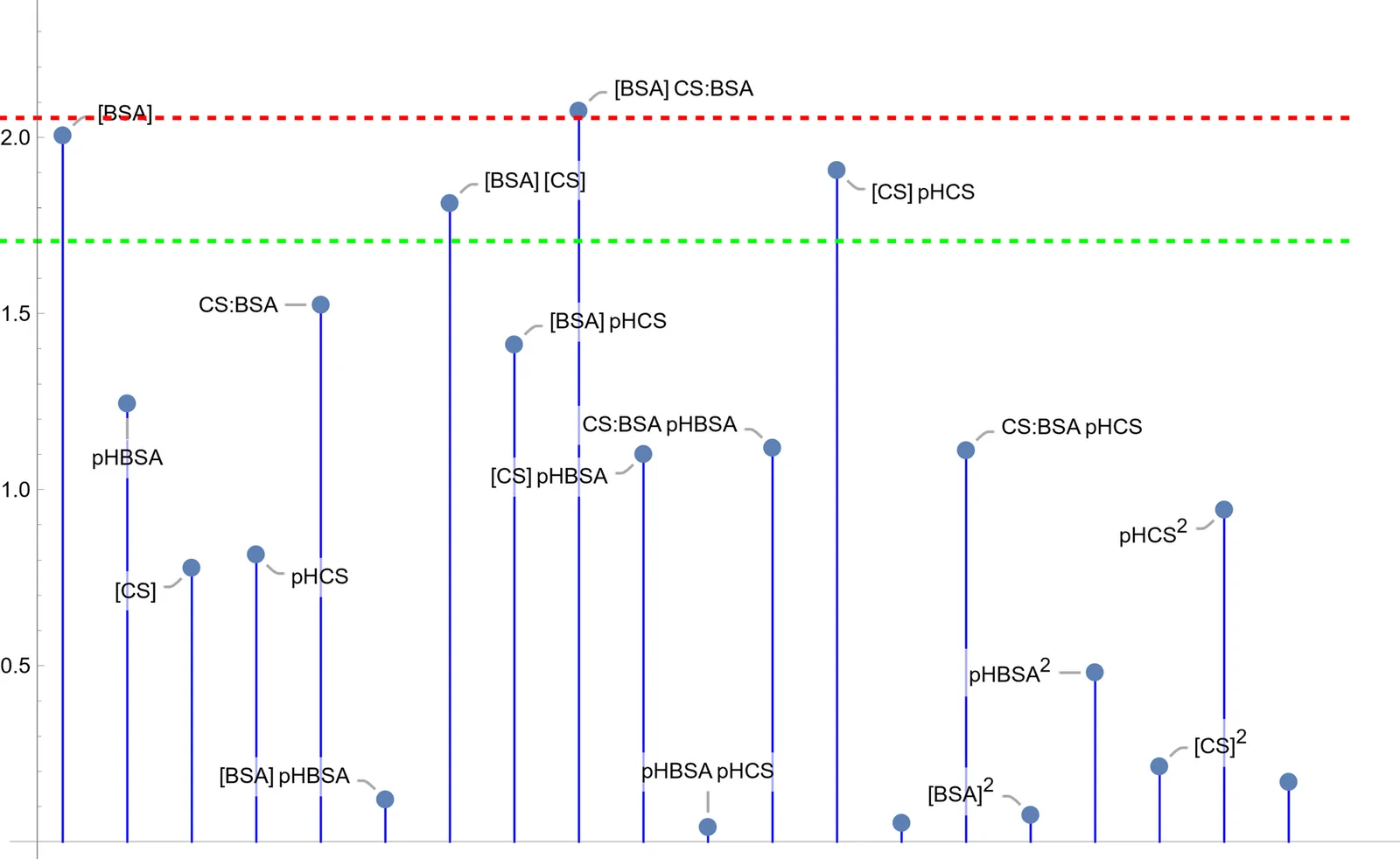
Parameter t-test absolute values for Size. Values over the green line are significant at 10% significance level, those above the red line are significant for 5% level of significance (coded factors)

As mentioned above, the different combinations of factors that affect the synthesis of BSA-CS NPs when using the ion gelation method produce very varied particle sizes, ranging from 66 to 1017 nm, so a correlation between this response variable and the significant factors seems to be very useful for its application in different fields. It should be noted that although the optimal particle size is usually below 200 nm to avoid the mononuclear phagocyte system, in certain situations (e.g. higher drug loadings, oral vaccines or gastrointestinal drug retention) can be desirable larger sizes [70–73]. Based on the parameter test of significance (Fig. 3), just the second-order interaction ‘[BSA] CS:BSA’ is significant at the 5% significance level. Nevertheless, in order to have a more coherent model, the corresponding main effects, that are between 5.55% (BSA concentration) and 13.94% (CS:BSA mass ratio) of significance level, are added to an initial model, which therefore would depend only on [BSA] and CS:BSA mass ratio, *Size* = *88.0617—17.960 CS:BSA—32.333 [BSA]* + *7.838 [BSA] CS:BSA*, explaining less than 20% of the data variability.

**Fig. 4:**
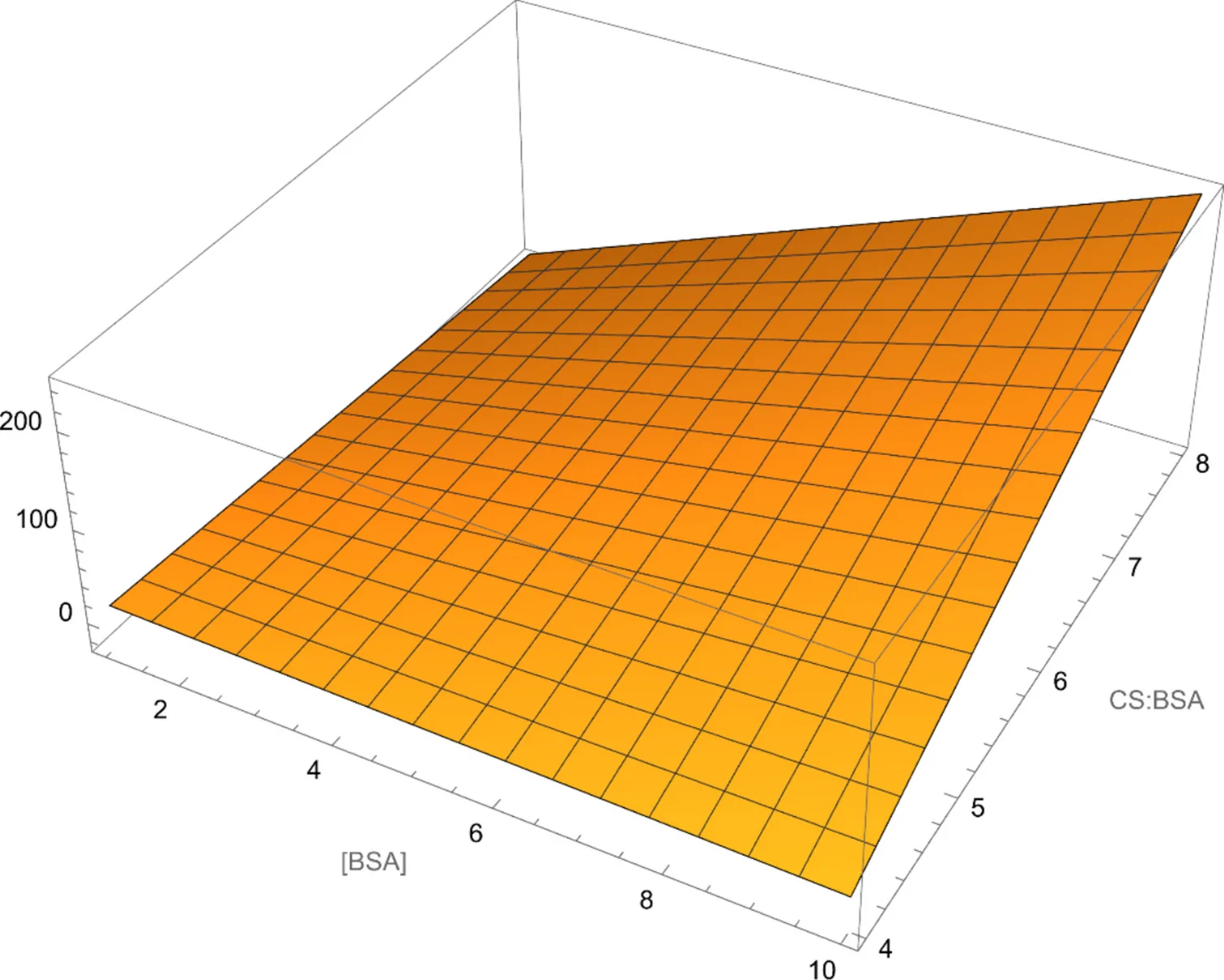
Final model for the response “size (nm)” as a function of ‘[BSA]’ (mg/ml) and ‘CS:BSA’ (mg:mg). Note that the axis of ‘CS:BSA’ represents the value for BSA (mg), with the value of CS being fixed at 1 mg

**Fig. 5:**
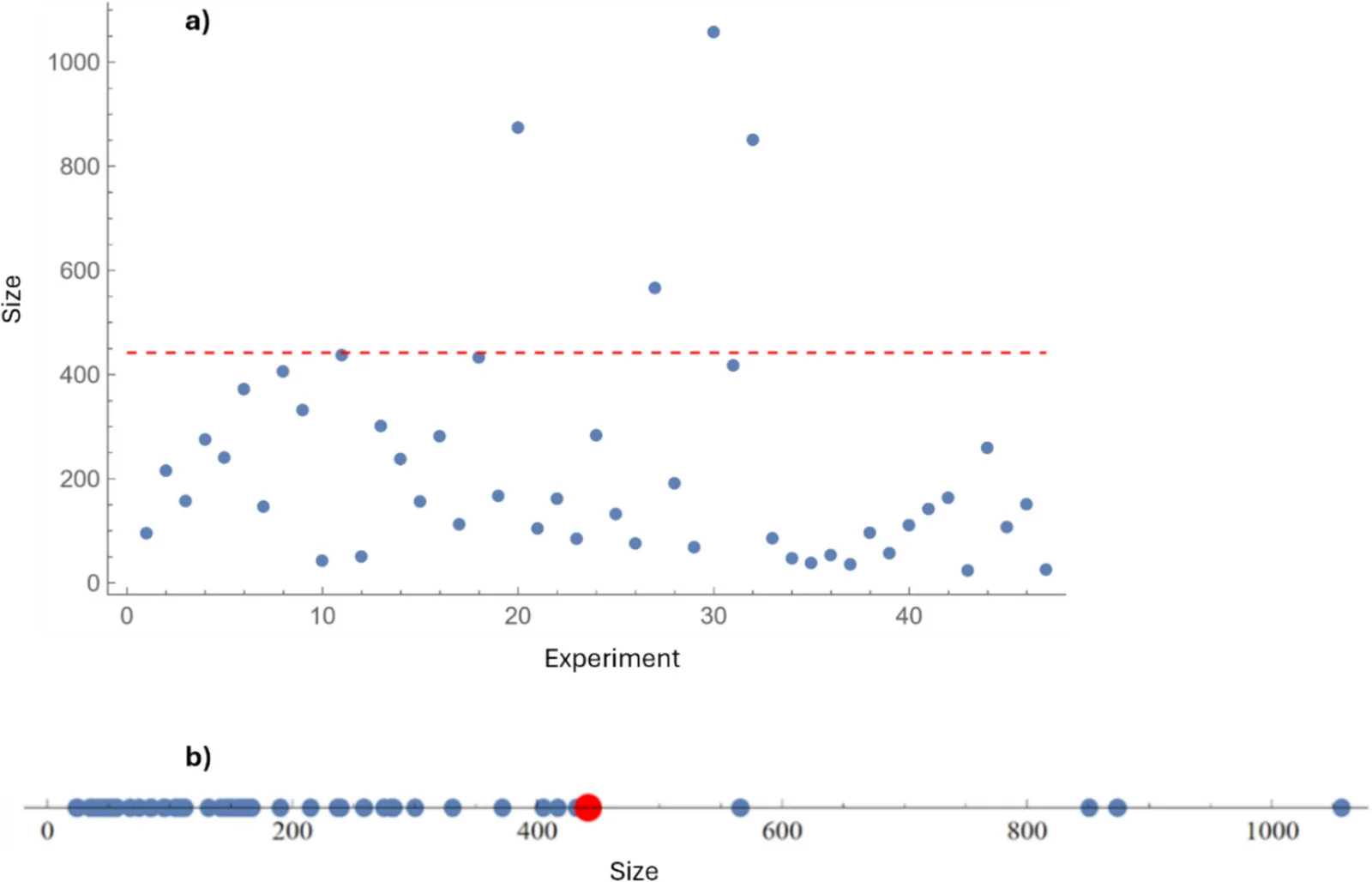
Values of the response “size (nm)” for the 47 experiments carried out with the ion gelation-based method. (**A**) The dashed line marks 442 nm; (**B**) The red dot shows 442 nm

As can be seen in Fig. 4, the maximal expected values of “Size “ from the initially proposed model is attained for big values of both main effects ‘[BSA]’ and ‘CS:BSA’, this mean size being close to 250 nm (right-top corner). Using the same initial model, the smallest expected values for this response (close to 0) are obtained for small values of ‘[BSA]’, regardless of the other factors. However, it should be taken into account that although the previous model is the best proposal from the statistical point of view, it is quite simple (without curvature since it does not contain quadratic terms), not reflecting the great variability of sizes that may appear within the experiments, always depending on the very often strong random component. In addition, note that the maximum discrepancy between data and the fitted model used to be at the borders of the design region. Then, to minimize the great differences between the experimental data and those predicted by the model, it seems convenient to add more parameters to the model, such as the following 3 most significant interactions (two of them between 5 and 10% of significance) and the most significant quadratic term to consider the curvature, which implies that the two main effects, ‘[CS]’ and ‘pHCS’ should be considered in the model as well. With these new additions, the maximum expected value for “size” is 442 nm, at the maximum levels of the 4 factors. Still, it seems to be quite different to the biggest values found at the experiments, but considering the random component and the discrepancy of a fitted model (specially at the borders of the design region) the distance is not that important, as it can be checked in Fig.5 (a and b), where it is clear that only 4 out of the 47 experiments resulted in mean sizes greater than 442 nm, showing that those 4 are quite apart from the majority of the observations and do not represent the general set of data.

Thus, after all the above considerations, the final proposed model for “Size” in natural variables is stated in Eq. (2), which is consistent with the limited existing literature on this method, which also does not perform statistical optimization [60, 61].

2 $$Size=1895.66-12.036 \;\left[BSA\right]-252.404\; \left[CS\right]-1386.13\; pHCS-17.960 \;CS:BSA+10.961\; \left[BSA\right] \;\left[CS\right]-16.658 \left[BSA\right] \;pHCS+7.838\; \left[BSA\right]\; CS:BSA+81.063\; \left[CS\right] \;pHCS+281.833 \;{pHCS}^{2}$$

where ‘[BSA]’ refers to BSA concentration, ‘CS:BSA’ to CS:BSA mass ratio, and ‘[CS]’ and ‘pHCS’ to chitosan concentration and pH, respectively, now explaining the 50% of the data variability (R^2^ = 0.496, Adjusted R^2^ = 0.374).

Regarding the minimum expected values for the mean size, there could appear negative sizes due to the deviations from the original data at the borders of the design region, which of course has no sense. In those cases, the predicted size should be taken as zero.

### Model for the response variable “ZP”

The ion gelation-based methodology enables the production of BSA-CS NPs with surface charge (ZP) values from neutral to positive depending on the relative quantities of both reactants (BSA and CS). Therefore, obtaining a correlation that allows predicting the values of this response variable when varying the levels of the significant factors can be very useful for the application of these particles in the transport of different molecules to different targets, considering their interaction with biological systems and barriers [74].

**Fig. 6:**
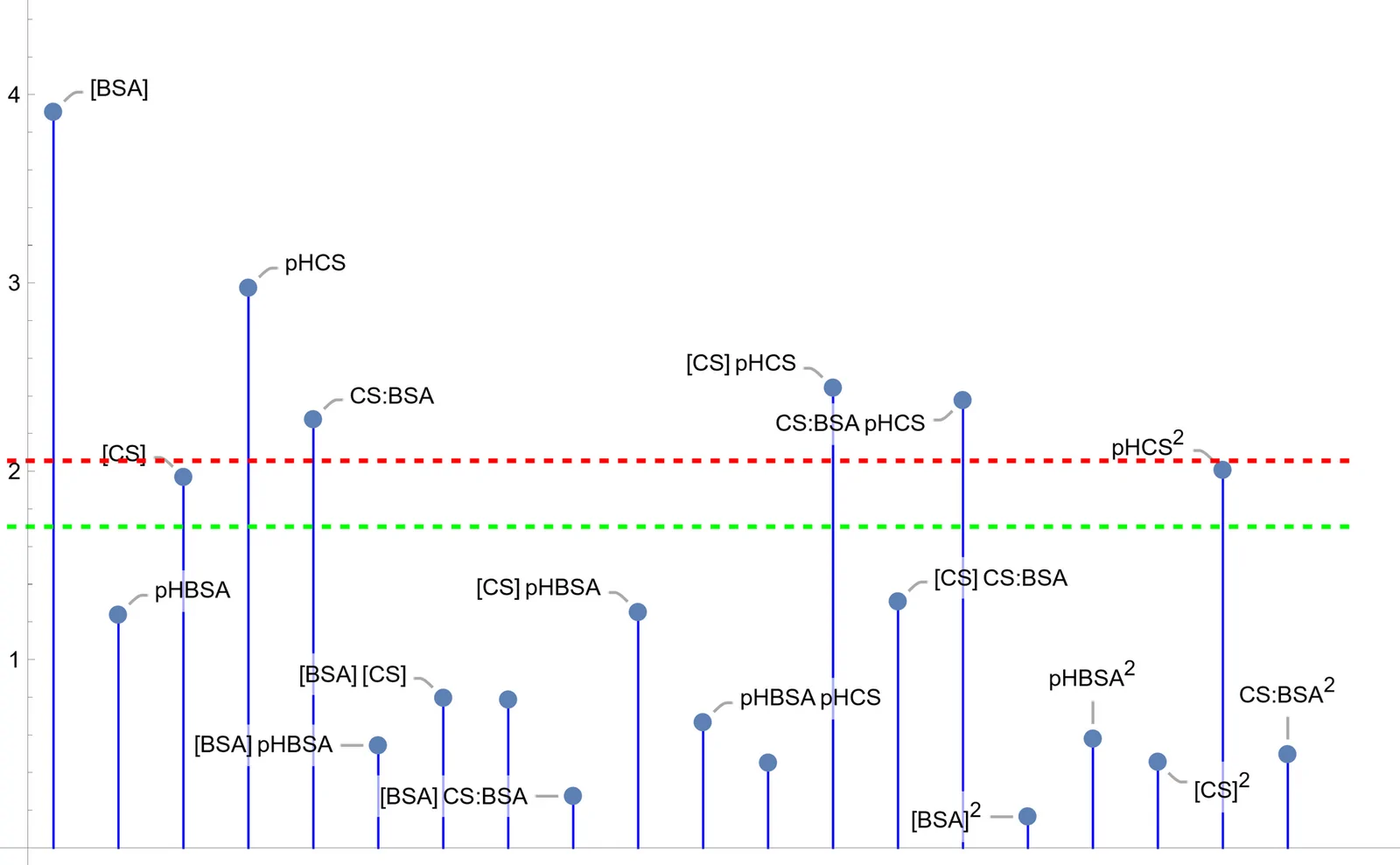
Parameter t-test absolute values for the “ZP” model (ion-gelation method). Values over the green line are significant at 10% significance level, those above the red line are significant for 5% level of significance

According to Fig. 6 (parameter test of significance) this response depends on several factors, in fact on all the 5 factors considered after the screening phase, but the pH of BSA. Note that although factors ‘[CS]’ and ‘pHCS^2^’ are between 5 and 10% significance level, they are very close to 5% and ‘[CS]’ is included in a significant interaction with the factor ‘pHCS’ (plus there is not any other factor in a similar situation). For all this, the final fitted model for the surface charge of NPs or ‘ZP’ is composed with the constant term, 4 main-effect terms, 2 two-factor interactions and a pure quadratic term, as is stated in Eq. (3), which explains the 61.6% of the data variability (R^2^ = 0.616, Adjusted R^2^ = 0.547). Without a doubt, the few authors who report on this synthesis method [61], together with studies on the complexation of CS with polyanions [75], support the results obtained in this work, that is, the pH of CS, the concentration of both reactants and the mass ratio between them, are the ones that seem to have the greatest effect on the surface charge of the NPs. Thus, since the values of the concentration of CS and BSA of this work, together with the mass ratios used, only allowed obtaining from neutral to positive surface charges, much higher mass ratios would be needed to obtain an inversion of charge [61].

3 $$ZP=-63.923+0.566 \;\left[BSA\right]+2.216 \;CS:BSA+3.834 \;\left[CS\right]+64.170\; pHCS-1.248\; CS:BSA \;pHCS-2.051 \;\left[CS\right] \;pHCS-11.841 \;{pHCS}^{2}$$

where ‘[BSA]’ refers to BSA concentration, ‘[CS]’ to CS concentration, ‘CS:BSA’ to CS:BSA mass ratio and ‘pHCS’ to CS pH.

From the model, the minimum and maximum expected values for ‘ZP’ are 3.185 and 23.921, for the values of the factors ‘[BSA]’, ‘[CS]’, ‘pHCS’ and ‘CS:BSA’ in natural variables {1, 3, 3, 1:8} and {10, 0.5, 2.5, 1:4}, respectively.

## Desolvation-based synthesis method with CS stabilization (BSA-CS_dsolv NPs)

### Model for the response variable “PDI”

**Fig. 7:**
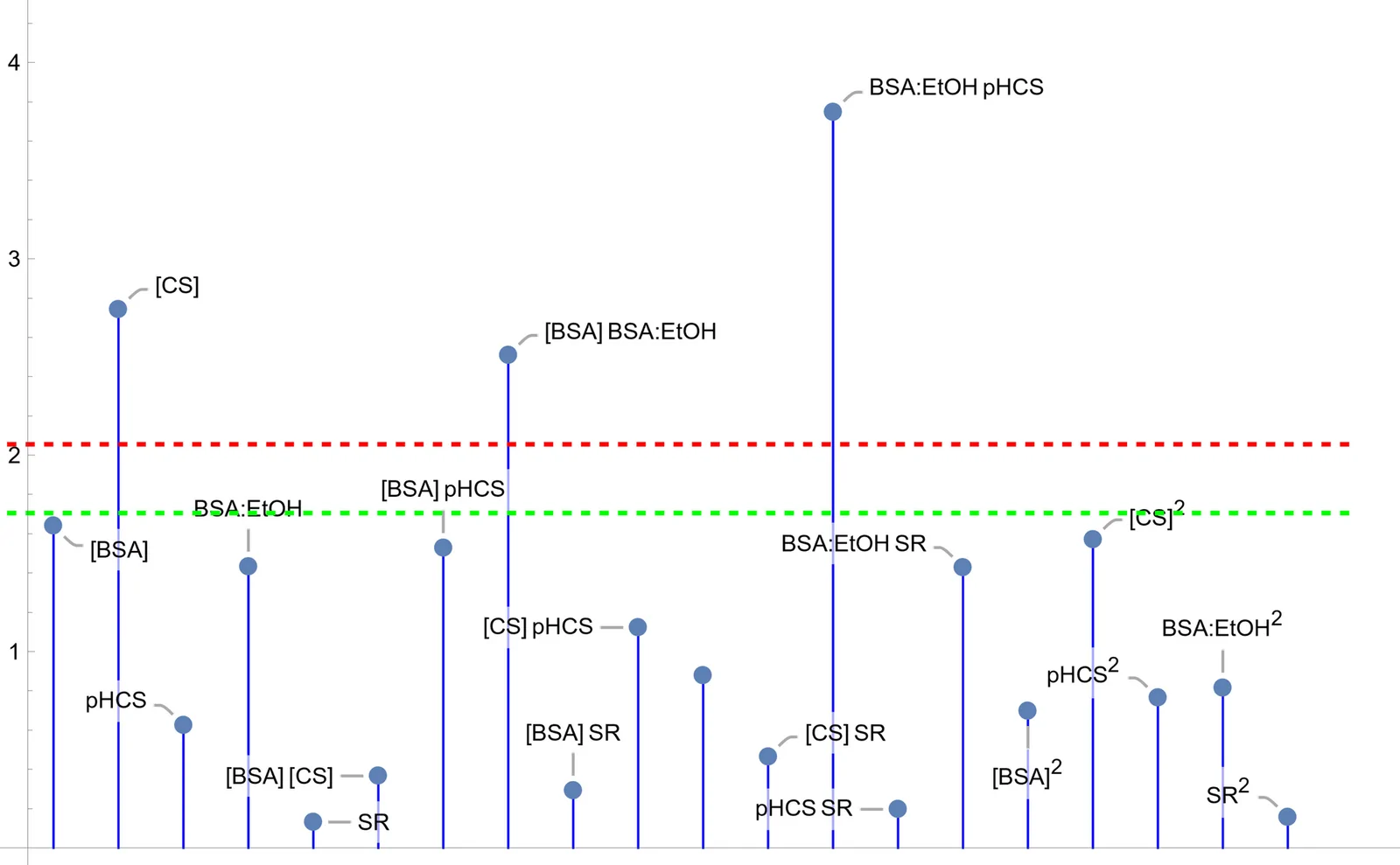
Parameter t-test absolute values for the ‘PDI’ model (desolvation-based method). Values over green line are significant at 10% significance level, those above the red line are significant for 5% level of significance

In the modified desolvation method reported in this work the stabilization of the generated BSA NPs occurs by the addition of a CS coating, so the mean PDI is shifted towards higher values than when using the ion gelation-based method. It appears that the efficiency of the nanoparticle coating process is behind the value of PDI. Moreover, nanoparticle aggregation can appear with incomplete electrostatic stabilization (partial CS coating) and/or heterogeneous coating kinetics [76, 77]. Certainly, obtaining a fitted function that allows determining the best combination of factors and levels to minimize this response is essential. In this regard, after applying the parameter test of significance (Fig. 7), two significant interactions (BSA:EtOH volume ratio with [BSA] and CS pH) and a main-effect, ‘[CS]’, which does not participate in the interactions, were found. However, when considering the model containing just these three parameters and the constant term, the expected minimum PDI value is an unacceptable 0.68. In order to reduce it to a more-desirable-values area, the model should be a bit more complex, adding terms that maybe statistically speaking are not so important, but they do are important to reduce the expected PDI. Then, after adding interactions and even a quadratic term, which are close to the 10% significance level, and the main effects [BSA], ‘pHCS’ and ‘BSA:EtOH’, each of one participating in two 2-interaction terms, we get a model that does not depend on the fifth main factor, ‘SR’, and allows obtaining expected mean values of PDI lower than 0.4 were obtained. Note that the calculus has been done using the codified factors (between −1 and 1), obtaining the initial models expressed in these factors, and then changing to natural variables (using the relationship X_i_ = (F_i_-c_i_)/d_i_, where F_i_ denotes the original factor, X_i_ is the corresponding coded one, c_i_ is the central point of the factor and d_i_ is the distance from the center to the extreme levels of the factor). Taking all the above into account, the final expression for the model is that described by Eq. (4), explaining 61.4% of the data variability (R^2^ = 0.614, Adjusted R^2^ = 0.532). As stated above, the material characteristics of the coating (CS concentration and pH) are critical parameters on PDI and, focusing solely on the conventional desolvation method [16, 67, 68], the BSA concentration and BSA:EtOH ratio should have a significant effect on PDI.

4 $$PDI=-1.027+0.013 \;\left[BSA\right]+0.302 \;\left[CS\right]+0.509 \;pHCS+0.615\; BSA:EtOH-0.00151 \;\left[BSA\right] BSA:EtOH-0.00235\; \left[BSA\right] \;pHCS-0.202\; BSA:EtOH \;pHCS-0.074 \;{\left[CS\right]}^{2}$$

where ‘[BSA]’ refers to BSA concentration, ‘[CS]’ to CS concentration, ‘BSA:EtOH’ to BSA:EtOH volume ratio and ‘pHCS’ to CS pH.

For the proposed model, the solutions that minimize the PDI values are in the borders (for instance {−1,−1,−0.829,−1,−1}, in coded variables) or even in the vertices of the design region, (e.g. {−1,−1,−1,−1,−1}, in coded variables), which could be not quite convenient as the conditions are more stressed. Thus, the best conditions to minimize PDI might be the first ones that in natural variables could be expressed as [BSA] = 10.0 mg/mL, [CS] = 0.5 mg/mL, ‘pHCS’ = 1.8 and ‘BSA:EtOH’ = 1:1 mL:mL (and the rest at the central levels). The expected PDI value for these conditions is 0.341 (note that the expected value in the vertex of the design region that has all the factors at the lower level is 0.313).

### Model for the response variable “size”

**Fig. 8:**
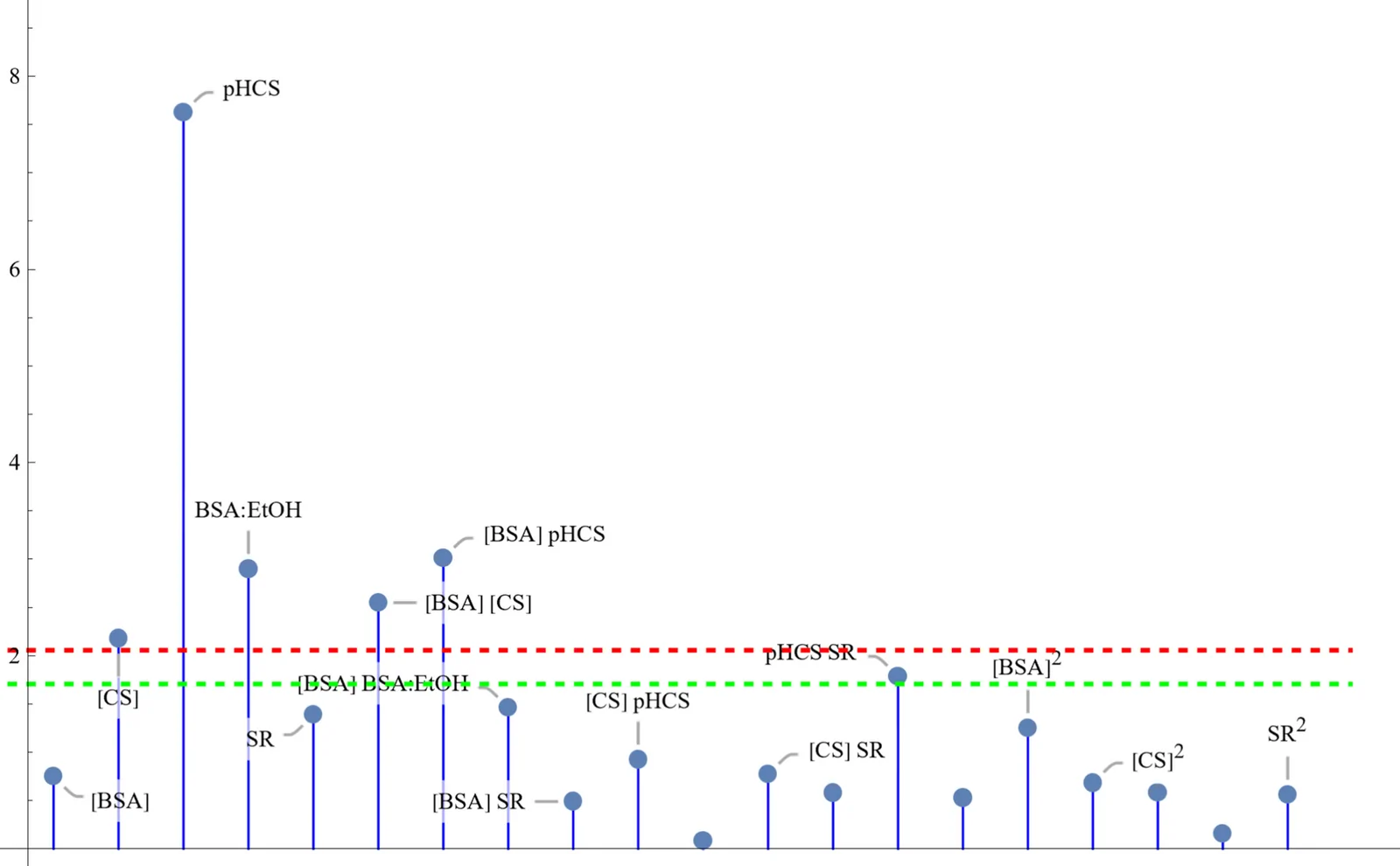
Parameter t-test absolute values for the “size” model (desolvation-based method). Values over green line are significant at 10% significance level, those above the red line are significant for 5% of significance level

Similar to what happens when applying the ion gelation-based method for BSA-CS NPs generation, the use of the modified desolvation methodology with CS allows obtaining sizes in a very wide range (37—1305 nm), so, in this case, it is also interesting to obtain a correlation between the mean size and the significant factors in the synthesis process, considering its importance for the rational design of the formulation to achieve the required therapeutic efficacy by its adequate biodistribution and cellular uptake. Thus, as it can be deduced from Fig. 8 (parameter test of significance), three main effects (concentration and pH of CS and BSA:EtOH volume ratio) and two two-factor interactions, [BSA] with CS concentration and pH, are significant at 5%. The factor ‘[BSA]’ appears in the two interactions, thus it seems sensible to keep the main effect in the model. Therefore, the final fitted model for the response variable “size” can be expressed by Eq. (5), which depends on all the main effects, except ‘SR’ (stirring rate of CS), and explains the 66% of the data variability (R^2^ = 0.660, Adjusted R^2^ = 0.609). The dependence of ‘[BSA]’ and ‘BSA:EtOH’ with the “size” is consistent with the existing literature on the conventional desolvation method, according to the mechanism of nucleation-and-growth to get nano-scale protein aggregates [78, 79]. Regarding the effect of the CS characteristics on the “size”, considering the lack of literature on the stabilization of the generated BSA NPs with CS, the significance found can be explained by the influence of the concentration of CS on the coating process and the pH of CS on the stabilization efficacy of the BSA NPs by the CS [47, 80].

5 $$Size=538.356+19.767\; \left[BSA\right]+97.612 \;BSA:EtOH+149.742\; \left[CS\right]-314.215 \;pHCS-2.023 \;\left[BSA\right] \;\left[CS\right]-7.655 \;\left[BSA\right] \;pHCS$$

where ‘[BSA]’ refers to BSA concentration, ‘[CS]’ to CS concentration, ‘BSA:EtOH’ to BSA:EtOH volume ratio and ‘pHCS’ to CS pH.

As has been mentioned repeatedly in this work, when looking for the extremes in a fitted model, the worst fitting is usually the closest to the borders of the design region. Taking this into account, the expected maximum for “size” from the model (above 800 nm), when taking ‘[BSA]’ and ‘BSA:EtOH’ at the maximum levels and [CS] and its pH at the minimum ones, should be considered with caution. Note that just 4 out of the 47 experiments carried out obtained values of size greater than 870 (those that are outliers and do not represent the majority of the data) with another 3 between 850 and 870, around which is the expected maximum. The same can be said about the minimum expected values, which are attained when ‘BSA:EtOH’ is taken at its minimum value and the other 3 at their maximum levels, getting an impossible negative expected value. In fact, for ‘[BSA]’, ‘[CS’] and ‘pHCS’ at their maximum values, the mean size depends linearly on ‘BSA:EtOH’. This relation should be between 1:2.5 and 1:3 to get coherent values of “size” (greater than zero), obtaining the expected minimum ones (close to 0) for the relation 1:2.5.

### Model for the response variable “ZP”

**Fig. 9:**
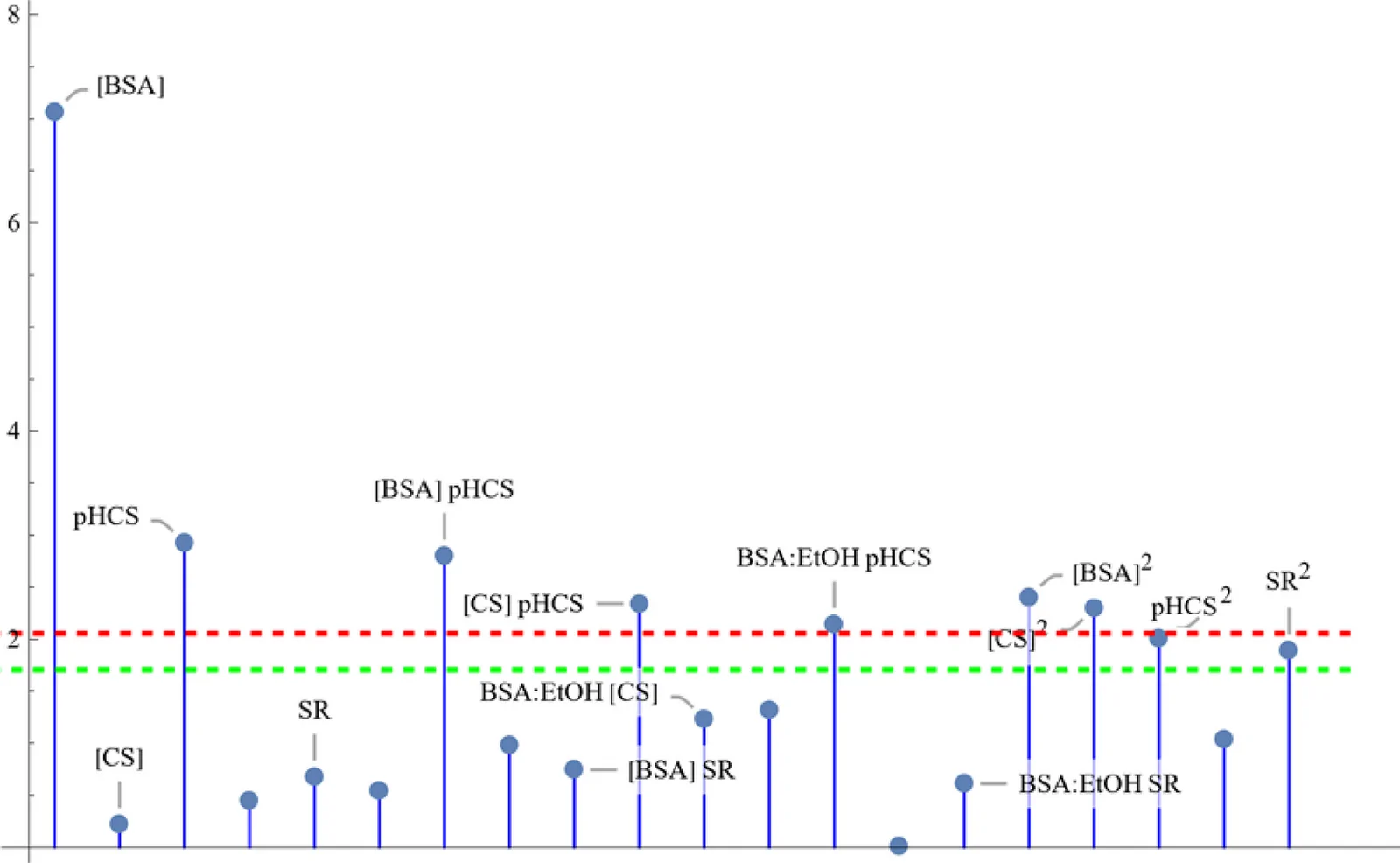
Parameter t-test absolute values for the “ZP” model (desolvation-based method). Values over green line are significant at 10% significance level, those above the red line are significant for 5% level of significance

As described above, the desolvation method with CS stabilization generates NPs with highly positive surface charges, with values greater than + 20 mV in most cases, due to the amine groups of the CS coating that stabilize the BSA NPs, which gives them superior performance in terms of cellular uptake [81–83], endosomal escape [84] or tumor penetration and tissue targeting [85–87]. Still, obtaining the relation between this response and the factors that affect the synthesis process can be useful. In this case, two main effects, ‘[BSA]’ and ‘pHCS’, three two-factor interactions, ‘[BSA] pHCS’, ‘[CS] pHCS’ and ‘BSA:EtOH pHCS’, and even two pure-quadratic terms (BSA and CS concentrations) are significant at the 5% significance level according to the parameter test of significance for the codified factors (Fig. 9).

Figure 9 shows a significance of 5.5% for the pure quadratic term ‘pHCS^2^’, very close to the limit, and since their main effect is significant and additionally it participates in every significant interaction, it seems convenient to keep this quadratic effect in the model, which together with the other two pure-quadratic terms indicates the possible existence of a strong curvature effect. This means that the shape of the model is far from being ‘flat’ (like in Fig. 4), but it may have “mountains” and “valleys”, which means that small variations of the effects that appear within the quadratic terms may produce noticeable changes of the response. On the other hand, the main effects ‘[CS]’ and ‘BSA:EtOH’ are maintained in the model due to their participating in interactions or even in quadratic effects. The final model does not depend on ‘SR’ and it has a small dependence on ‘BSA:EtOH’, just through the interaction with ‘pHCS’ that is in the limit of significance (p = 0.0415). As a matter of fact, ‘pHCS’ seems to be quite influential in the model since it appears in the 3 interactions, as well as a main and pure quadratic effects. Thus, everything mentioned above is contemplated in the terms of Eq. (6). Note that the surface charge of these NPs depends entirely on the protonation state of CS since this biopolymer stabilizes the BSA NPs with a coating, which demonstrates the predominant effect of ‘pHCS’ on ZP [88]. Obviously, the other determining significant factor in this synthesis process is the concentration of CS, whose molecules are absorbed onto the surface of the negatively charged BSA NPs by electrostatic interaction, but with saturation plateau relative to the ZP, generating inter-molecular repulsive forces that stabilizes the BSA NPs [89, 90]. Given that [BSA] and ‘BSA:EtOH’ primarily affect the formation of the BSA NPs and that the desolvation method is a physical process not a chemical reaction, it seems reasonable to assume the limited influence of these factors over the ZP [91–93], contradicting what has been found here regarding [BSA]. However, it should be noted that, to the best of our knowledge, there is no literature specifically related to the synthesis process under discussion.

6 $$ZP=-250.114-0.390\; \left[BSA\right]+28.349 \;\left[CS\right]+234.002 \;pHCS+11.502 \;BSA:EtOH-0.203\; \left[BSA\right] \;pHCS-4.736 \;\left[CS\right] \;pHCS-5.426\; pHCS\ BSA:EtOH+0.00696 \;{\left[BSA\right]}^{2}-5.231 \;{\left[CS\right]}^{2}-46.831\; {pHCS}^{2}$$

where ‘[BSA]’ refers to BSA concentration, ‘[CS]’ to CS concentration, ‘BSA:EtOH’ to BSA:EtOH volume ratio and ‘pHCS’ to CS pH.

From the model, which explains almost the 72% of the data variability (R^2^ = 0.7195, Adjusted R^2^ = 0.642), the minimum and maximum expected values for “ZP” are 9.523 and 30.592 for the values of [BSA], [CS], pHCS and BSA:EtOH of {64.5, 3.0, 2.5, 1:3} and {80.0, 1.7,2.2, 1:1}, respectively.

## Conclusions

In this article, two synthesis approaches of albumin-based nanoparticles have been mathematically optimized by a two-step design of experiments methodology. These methods of synthesis do not use reducing/denaturant compounds, organic solvents, high temperatures or high-shear homogenization like traditional methods, using CS as a stabilizer. In particular, CS was used to apply the ionic gelation method in the generation of albumin particles by electrostatic interactions and to add a stabilizing CS coating to desolvation-generated albumin nanoparticles. The DoE methodology applied in this work consisted of a first screening step to detect the most influential factors of the synthesis processes on the physicochemical properties of the albumin-CS nanoparticles by 2_IV_^8−4^ or 2_IV_^7−3^ fractional designs. Then, the selected factors were subjected to a Response Surface Methodology (RSM) using a Central Composite Design (CCD), resulting in fitted models for the response variables (size, polydispersity index or PDI and surface charge). Using this DoE-based approach, the best conditions to minimize the PDI and the combination of factors and levels to obtain the minimum and maximum expected values for the mean size and surface charge of the nanoparticles were determined for both synthesis methods.

The use of the ion gelation method to obtain albumin nanoparticles by the electrostatic interaction between the negatively charged protein at basic pH and the positively charged CS at acidic conditions allowed to obtain a very wide range of sizes (66 to 1017 nm), but with PDI mean values between 0.3 and 0.6, with those below 0.4 being acceptable, and surface charge values from neutral to positive (20 mV). Eight factors with possible influence on this synthesis procedure were selected from pilot experiments and a literature review, which were reduced to 5 after applying the screening procedure. When applying the RSM with CCD phase to these factors, taking into account the parameter tests of significance, the fitted models for all the response variables were obtained, which depend mainly on four factors, albumin and chitosan concentrations, pH of chitosan and chitosan:albumin mass ratio. According to the proposed models, the solution that minimize PDI to a value of 0.363 is [BSA] = 2.28 mg/ml; [CS] = 1.43 mg/ml, pH of CS = 2.2 and CS:albumin mass ratio = 1:7.3 (mg:mg), the remaining factors being fixed at the central levels, and the expected maximum for size (442 nm) is achieved at the maximum levels of the four factors. Regarding the surface charge, the minimum and maximum expected values are 3.19 and 23.9 mV for the values of the factors [BSA] (mg/ml), [CS] (mg/ml), pH of CS and CS:BSA (mg:mg) of {1, 3, 3, 1:8} and {10, 0.5, 2.5, 1:4}, respectively.

The modification of the traditional desolvation method, using CS as a stabilizing coating, allows to avoid the use of toxic crosslinking agents, such as glutaraldehyde, but increases the mean PDI values up to 0.4—0.7. Nevertheless, the size of the albumin nanoparticles generated with this alternative synthesis approach can be easily modified from 37 to 1305 nm and the colloidal suspension obtained is very stable with highly positive surface charge values (20—40 mV) in most cases. For this synthesis method, fitted models for the response variables were also obtained by applying the RSM with CCD to the five influential factors that remained after subjecting the seven factors initially considered to the screening procedure. All responses depended on 4 main effects (albumin and CS concentrations, pH of CS and albumin:ethanol volume ratio). The best combination of factors to obtain a minimum value of PDI (0.341) from the final fitted models is [BSA] = 10.0 mg/ml, [CS] = 0.5 mg/ml, pH of CS = 1.8 and BSA:EtOH volume ratio = 1:1 mL:mL (and the rest of factors at their central levels). Regarding the size, the maximum expected (above 800 nm) can be attained when the [BSA] and the BSA:EtOH volume ratio are at their maximum levels and the [CS] and its pH are at the minimum ones. To achieve the minimum values, [BSA], [CS] and their pH should be at their maximum values, but the BSA:EtOH volume ratio should be between 1:2.5 and 1:3.0 to obtain values above cero (1:2.5 to obtain a value close to 0). Finally, the minimum and maximum expected values for the surface charge from the fitted model, 9.5 and 30.6 mV, can be obtained with the values of [BSA] (mg/ml), [CS] (mg/ml), ‘pHCS’ and ‘BSA:EtOH’ (mL:mL) of {64.5, 3.0, 2.5, 1:3} and {80.0, 1.7,2.2, 1:1}, respectively.

Table 11 shows a summary of the factors with influence in both synthesis processes for the three response variables, according to the final fitted models proposed in this work, along with the combinations of factor values that would produce the optimal values of each response. It should be noted that the response variable that controls the choice of the optimal formulation of each synthesis process is the PDI since the stability, reproducibility, controlled drug release and biological interactions of the NPs depends on it to a greater extent. Thus, the conditions that minimize PDI in the ion gelation-based method would yield a mean size of 27.7 nm, below 50 nm that is the preferred typical minimum value, although the threshold value to avoid renal clearance is usually 20 nm, and a surface charge of 16.4 mV. Regarding the desolvation-based method, when using the predicted conditions to obtain the minimum PDI value, the expected value for the mean size would be 146.2 nm, which is within the typical range of sizes for conventional administration routes, and for the surface charge would be 29.5 mV, a value very close to the maximum predicted, which give them a superior in vivo performance.

**Table 11:** Influential factors initially considered and in the final fitted models for the three response variables in the two synthesis processes considered in this work, as well as optimality conditions for each case. In the cells of the response variables corresponding to the ‘Model’, the significant effects of the final model are shown (main effects in first row, interactions in second row and quadratic effects in the third row). In the cells corresponding to ‘Optim. cond.’ appear the values of the factors that remain in the model that would lead to the optimal values of the responses

Synthesis methods and influential factors		Response variables		
		PDI	Size	ZP
**Ion gelation-based method** Initially considered factors: x[I]: [BSA]; x[II]: BSA pH; x[III]: [CS]; x[IV]: CS pH; x[V]: CS:BSA mass ratio; x[VI]: BSA flow rate; x[VII] stirring rate (addition of BSA over CS); x[VIII]: stirring rate post addition Factors in the fitted models: [BSA] mg/mL (1), [CS] mg/mL (2), CS pH (3), CS:BSA mL:mL (4)	Model	1, 2, 3, 4 1*2, 2*3, 3*4 3^2^	1, 2, 3, 4 1*2, 1*3, 1*4, 2*3 3^2^	1, 2, 3, 4 2*3, 3*4 3^2^
	Optim. cond	{2.3,1.4,2.2,1:7.3} **(min)** PDI: 0.363	{10,3,3,1:8} (max) {10,3,2.05,1:8} (min)	{1,3,3,1:8} (max) {10,0.5,2.5,1:4} (min)
**Desolvation-based method** Initially considered factors: x[I]: [BSA]; x[II]: BSA pH; x[III]: [CS]; x[IV]: CS pH; x[V]: BSA:EtOH volume ratio; x[VI]: EtOH flow rate; x[VII] stirring rate Factors in the fitted models: [BSA] mg/mL (1), [CS] mg/mL (2), CS pH (3), BSA:EtOH mL:mL (4)	Model	1, 2, 3, 4 1*3, 1*4, 3*4 2^2^	1, 2, 3, 4 1*2, 1*3	1, 2, 3, 4 1*3, 2*3, 3*4 1^2^, 2^2^, 3^2^
	Optim. cond	{10,0.5,1.8,1:1} **(min)** PDI: 0.341	{80,0.5,1.7,1:3 (max) {80,3,2.5,1:2.5} (min)	{64.5, 3.0, 2.5, 1:3} (max) {80.0, 1.7,2.2, 1:1} (min)

Throughout this article, a great variety of designs have been employed, which include full factorial, fractional factorial and non-rotatable central composite designs. However, in view of an extension of this work, the application of the optimal experimental design theory [94] would be an interesting approach that might complement the results obtained here. Nevertheless, when using this approach, it should be considered that in the study there are several responses to be observed, that is, we are dealing with multiresponse models and a correlation structure between the response variables should be taken into account [95, 96]. Moreover, in this work, two different synthesis processes or ‘blocks’ have been optimized, adding a new correlation layer to the structure, a case that has been treated in the literature [97].

## Supplementary information

Below is the link to the electronic supplementary material.

## Data Availability

The relevant data that supports the results of the present paper are included within the manuscript.
