# Supplementary material for: Statistical optimization of chitosan-based synthesis strategies to generate albumin nanoparticles
Source: Drug Deliv Transl Res. 2026 Feb 16;16(9):3381–404. doi: 10.1007/s13346-026-02046-4 (PMC13538268; doi:10.1007/s13346-026-02046-4)
Supplement: Supplementary file 1 [file 13346_2026_2046_MOESM1_ESM.docx]

Appendix. Detailed results of the Screening Procedure

**ION GELATION-BASED SYNTHESIS METHOD (BSA-CS_ie NPs).**

Results performed with SPSS v.22 software for the codified variables X1,…,X8, coming respectively from [BSA], pH BSA, [CS], pH CS, CS:BSA, BSA FR, SR (during), SR (post), first taking just the 16 experiments of the fractional design, and then when considering the preliminary experiments as well.

The variables significant at 5% are highlighted in green, and the ones significant at 10% are highlighted in blue. From the results, it seems that X1, X2, X3, X4 and X5 may be important for at least one response, discarding X6, X7 and X8. Anyway, let us note that these three took a constant value for the preliminary experiments and thus those data did not provide information about those factors’ influence.

| Size (fractional design) | | | | | |
| --- | --- | --- | --- | --- | --- |
| Source | Sum of Squares | DF | Mean Square | F ratio | Significance (p value) |
| X1 | 98949.566 | 1 | 98949.566 | 2.116 | 0.189 |
| X2 | 105408.986 | 1 | 105408.986 | 2.254 | 0.177 |
| X3 | 8544.691 | 1 | 8544.691 | 0.183 | 0.682 |
| X4 | 1500.594 | 1 | 1500.594 | 0.032 | 0.863 |
| X5 | 371.237 | 1 | 371.237 | 0.008 | 0.931 |
| X6 | 72542.689 | 1 | 72542.689 | 1.551 | 0.253 |
| X7 | 14073.670 | 1 | 14073.670 | 0.301 | 0.600 |
| X8 | 3990.133 | 1 | 3990.133 | 0.085 | 0.779 |
| Error | 327327.510 | 7 | 46761.073 |  |  |
| Total | 632709.076 | 15 |  |  |  |

| Size (fractional design + preliminary experiments) | | | | | |
| --- | --- | --- | --- | --- | --- |
| Source | Sum of Squares | DF | Mean Square | F ratio | Significance (p value) |
| X1 | 138302.226 | 1 | 138302.226 | 3.219 | 0.081 |
| X2 | 139018.137 | 1 | 139018.137 | 3.236 | 0.080 |
| X3 | 73388.354 | 1 | 73388.354 | 1.708 | 0.199 |
| X4 | 857.929 | 1 | 857.929 | 0.020 | 0.888 |
| X5 | 34735.970 | 1 | 34735.970 | 0.809 | 0.374 |
| X6 | 43582.914 | 1 | 43582.914 | 1.015 | 0.320 |
| X7 | 1435.031 | 1 | 1435.031 | 0.033 | 0.856 |
| X8 | 625.480 | 1 | 625.480 | 0.015 | 0.905 |
| Error | 1675375.984 | 39 | 42958.359 |  |  |
| Total | 2122650.302 | 47 |  |  |  |

| PDI (fractional design) | | | | | | |
| --- | --- | --- | --- | --- | --- | --- |
| Source | Sum of Squares | DF | Mean Square | F ratio | Significance (p value) | |
| X1 | 0.002 | 1 | 0.002 | 0.028 | 0.873 | |
| X2 | 0.030 | 1 | 0.030 | 0.347 | 0.574 | |
| X3 | 0.097 | 1 | 0.097 | 1.112 | 0.327 | |
| X4 | 0.146 | 1 | 0.146 | 1.677 | 0.236 | |
| X5 | 0.001 | 1 | 0.001 | 0.007 | 0.938 | |
| X6 | 0.106 | 1 | 0.106 | 1.211 | 0.308 | |
| X7 | 0.002 | 1 | 0.002 | 0.022 | 0.887 | |
| X8 | 0.003 | 1 | 0.003 | 0.038 | 0.851 | |
| Error | 0.611 | 7 | 0.087 |  |  | |
| Total | 0.998 | 15 |  |  |  | |
| PDI (fractional design + preliminary experiments) | | | | | | |
| Source | Sum of Squares | DF | Mean Square | F ratio | | Significance (p value) |
| X1 | 0.082 | 1 | 0.082 | 1.680 | | 0.203 |
| X2 | 0.004 | 1 | 0.004 | 0.075 | | 0.786 |
| X3 | 0.050 | 1 | 0.050 | 1.029 | | 0.317 |
| X4 | 0.267 | 1 | 0.267 | 5.445 | | 0.025 |
| X5 | 9.352E-5 | 1 | 9.352E-5 | 0.002 | | 0.965 |
| X6 | 0.060 | 1 | 0.060 | 1.230 | | 0.274 |
| X7 | 0.030 | 1 | 0.030 | 0.607 | | 0.440 |
| X8 | 0.003 | 1 | 0.003 | 0.069 | | 0.795 |
| Error | 1.909 | 39 | 0.049 |  | |  |
| Total | 2.385 | 47 |  |  | |  |

| ZP (fractional design) | | | | | |
| --- | --- | --- | --- | --- | --- |
| Source | Sum of Squares | DF | Mean Square | F ratio | Significance (p value) |
| X1 | 385.268 | 1 | 385.268 | 16.447 | 0.005 |
| X2 | 6.725 | 1 | 6.725 | 0.287 | 0.609 |
| X3 | 130.605 | 1 | 130.605 | 5.575 | 0.050 |
| X4 | 62.857 | 1 | 62.857 | 2.683 | 0.145 |
| X5 | 112.217 | 1 | 112.217 | 4.790 | 0.065 |
| X6 | 15.431 | 1 | 15.431 | 0.659 | 0.444 |
| X7 | 72.365 | 1 | 72.365 | 3.089 | 0.122 |
| X8 | 0.499 | 1 | 0.499 | 0.021 | 0.888 |
| Error | 163.978 | 7 | 23.425 |  |  |
| Total | 949.946 | 15 |  |  |  |
| ZP (fractional design + preliminary experiments) | | | | | |
| Source | Sum of Squares | DF | Mean Square | F ratio | Significance (p value) |
| X1 | 48.892 | 1 | 48.892 | 0.140 | 0.710 |
| X2 | 505.857 | 1 | 505.857 | 1.450 | 0.236 |
| X3 | 9.832 | 1 | 9.832 | 0.028 | 0.868 |
| X4 | 993.392 | 1 | 993.392 | 2.847 | 0.100 |
| X5 | 4.492 | 1 | 4.492 | 0.013 | 0.910 |
| X6 | 70.943 | 1 | 70.943 | 0.203 | 0.655 |
| X7 | 32.231 | 1 | 32.231 | 0.092 | 0.763 |
| X8 | 10.031 | 1 | 10.031 | 0.029 | 0.866 |
| Error | 13605.827 | 39 | 348.867 |  |  |
| Total | 15274.467 | 47 |  |  |  |

**DESOLVATION-BASED SYNTHESIS METHOD WITH CS STABILIZATION (BSA-CS_dsolv NPs).**

Results performed with SPSS v.22 software for the codified variables X1,…,X7, coming respectively from [BSA], pH BSA, [CS], pH CS, BSA:EtOH, EtOH FR and SR, first taking just the 16 experiments of the fractional design, and then when considering the preliminary experiments as well. The variables significant at 5% are highlighted in green, and the ones significant at 10% are highlighted in blue. From the results, it seems that X1, X3, X4, X5 and X7 may be important for at least one response, discarding X2 and X6. Anyway, let us note that X6 and X7 maintained constant value for the preliminary experiments and thus those data did not provide information about these factors’ influence.

| Size (fractional design) | | | | | |
| --- | --- | --- | --- | --- | --- |
| Source | Sum of Squares | DF | Mean Square | F ratio | Significance (p value) |
| X1 | 90.250 | 1 | 90.250 | 0.001 | 0.973 |
| X2 | 11481.123 | 1 | 11481.123 | 0.152 | 0.707 |
| X3 | 68016.640 | 1 | 68016.640 | 0.900 | 0.371 |
| X4 | 491891.822 | 1 | 491891.822 | 6.510 | 0.034 |
| X5 | 136419.423 | 1 | 136419.423 | 1.805 | 0.216 |
| X6 | 90.250 | 1 | 90.250 | 0.001 | 0.973 |
| X7 | 110789.123 | 1 | 110789.123 | 1.466 | 0.260 |
| Error | 604483.587 | 8 | 75560.448 |  |  |
| Total | 1423262.218 | 15 |  |  |  |

| Size (fractional design + preliminary experiments) | | | | | |
| --- | --- | --- | --- | --- | --- |
| Source | Sum of Squares | DF | Mean Square | F ratio | Significance (p value) |
| X1 | 81650.252 | 1 | 81650.252 | 1.534 | 0.223 |
| X2 | 41556.339 | 1 | 41556.339 | 0.781 | 0.382 |
| X3 | 293321.981 | 1 | 293321.981 | 5.512 | 0.024 |
| X4 | 2422670.535 | 1 | 2422670.535 | 45.525 | 0.000 |
| X5 | 953808.660 | 1 | 953808.660 | 17.923 | 0.000 |
| X6 | 67463.325 | 1 | 67463.325 | 1.268 | 0.267 |
| X7 | 15093.559 | 1 | 15093.559 | 0.284 | 0.597 |
| Error | 2128629.215 | 40 | 53215.730 |  |  |
| Total | 5989528.309 | 47 |  |  |  |

| PDI (fractional design) | | | | | |
| --- | --- | --- | --- | --- | --- |
| Source | Sum of Squares | DF | Mean Square | F ratio | Significance (p value) |
| X1 | 0.007 | 1 | 0.007 | 0.171 | 0.690 |
| X2 | 0.001 | 1 | 0.001 | 0.034 | 0.859 |
| X3 | 0.180 | 1 | 0.180 | 4.727 | 0.061 |
| X4 | 0.009 | 1 | 0.009 | 0.228 | 0.646 |
| X5 | 0.000 | 1 | 0.000 | 0.006 | 0.942 |
| X6 | 0.054 | 1 | 0.054 | 1.416 | 0.268 |
| X7 | 0.015 | 1 | 0.015 | 0.402 | 0.544 |
| Error | 0.305 | 8 | 0.038 |  |  |
| Total | 0.571 | 15 |  |  |  |

| PDI (fractional design + preliminary experiments) | | | | | |
| --- | --- | --- | --- | --- | --- |
| Source | Sum of Squares | DF | Mean Square | F ratio | Significance (p value) |
| X1 | 0.006 | 1 | 0.006 | 0.251 | 0.619 |
| X2 | 0.000 | 1 | 0.000 | 0.004 | 0.947 |
| X3 | 0.174 | 1 | 0.174 | 6.832 | 0.013 |
| X4 | 0.070 | 1 | 0.070 | 2.733 | 0.106 |
| X5 | 0.030 | 1 | 0.030 | 1.182 | 0.283 |
| X6 | 0.039 | 1 | 0.039 | 1.526 | 0.224 |
| X7 | 0.050 | 1 | 0.050 | 1.971 | 0.168 |
| Error | 1.019 | 40 | 0.025 |  |  |
| Total | 1.363 | 47 |  |  |  |

| ZP (fractional design) | | | | | |
| --- | --- | --- | --- | --- | --- |
| Source | Sum of Squares | DF | MS | F ratio | Significance (p value) |
| X1 | 243.360 | 1 | 243.360 | 8.802 | 0.018 |
| X2 | 48.303 | 1 | 48.303 | 1.747 | 0.223 |
| X3 | 17.640 | 1 | 17.640 | 0.638 | 0.447 |
| X4 | 57.760 | 1 | 57.760 | 2.089 | 0.186 |
| X5 | 3.422 | 1 | 3.422 | 0.124 | 0.734 |
| X6 | 1.322 | 1 | 1.322 | 0.048 | 0.832 |
| X7 | 171.610 | 1 | 171.610 | 6.207 | 0.037 |
| Error | 221.193 | 8 | 27.649 |  |  |
| Total | 764.610 | 15 |  |  |  |

| ZP (fractional design + preliminary experiments) | | | | | |
| --- | --- | --- | --- | --- | --- |
| Source | Sum of Squares | DF | MS | F ratio | Significance (p value) |
| X1 | 1612.401 | 1 | 1612.401 | 64.374 | 0.000 |
| X2 | 4.201 | 1 | 4.201 | 0.168 | 0.684 |
| X3 | 12.403 | 1 | 12.403 | 0.495 | 0.486 |
| X4 | 287.141 | 1 | 287.141 | 11.464 | 0.002 |
| X5 | 55.901 | 1 | 55.901 | 2.232 | 0.143 |
| X6 | 67.902 | 1 | 67.902 | 2.711 | 0.107 |
| X7 | 74.314 | 1 | 74.314 | 2.967 | 0.093 |
| Error | 1001.892 | 40 | 25.047 |  |  |
| Total | 3075.590 | 47 |  |  |  |
